# Bioprinting of human pluripotent stem cell derived corneal endothelial cells with hydrazone crosslinked hyaluronic acid bioink

**DOI:** 10.1186/s13287-024-03672-w

**Published:** 2024-03-14

**Authors:** Pyry Grönroos, Anni Mörö, Paula Puistola, Karoliina Hopia, Maija Huuskonen, Taina Viheriälä, Tanja Ilmarinen, Heli Skottman

**Affiliations:** 1 Faculty of Medicine and Health Technology, Tampere University, Arvo Ylpön Katu 34, 33520 Tampere, Finland; 2 Tays Eye Centre, Tampere University Hospital, Tampere, Finland

**Keywords:** Bioprinting, Bioink, Cornea, Human pluripotent stem cells, Corneal endothelial cells

## Abstract

**Background:**

Human corneal endothelial cells lack regenerative capacity through cell division in vivo*.* Consequently, in the case of trauma or dystrophy, the only available treatment modality is corneal tissue or primary corneal endothelial cell transplantation from cadaveric donor which faces a high global shortage. Our ultimate goal is to use the state-of-the-art 3D-bioprint technology for automated production of human partial and full-thickness corneal tissues using human stem cells and functional bioinks. In this study, we explore the feasibility of bioprinting the corneal endothelium using human pluripotent stem cell derived corneal endothelial cells and hydrazone crosslinked hyaluronic acid bioink.

**Methods:**

Corneal endothelial cells differentiated from human pluripotent stem cells were bioprinted using optimized hydrazone crosslinked hyaluronic acid based bioink. Before the bioprinting process, the biocompatibility of the bioink with cells was first analyzed with transplantation on ex vivo denuded rat and porcine corneas as well as on denuded human Descemet membrane. Subsequently, the bioprinting was proceeded and the viability of human pluripotent stem cell derived corneal endothelial cells were verified with live/dead stainings. Histological and immunofluorescence stainings involving ZO1, Na^+^/K^+^-ATPase and CD166 were used to confirm corneal endothelial cell phenotype in all experiments. Additionally, STEM121 marker was used to identify human cells from the ex vivo rat and porcine corneas.

**Results:**

The bioink, modified for human pluripotent stem cell derived corneal endothelial cells successfully supported both the viability and printability of the cells. Following up to 10 days of ex vivo transplantations, STEM121 positive cells were confirmed on the Descemet membrane of rat and porcine cornea demonstrating the biocompatibility of the bioink. Furthermore, biocompatibility was validated on denuded human Descemet membrane showing corneal endothelial -like characteristics. Seven days post bioprinting, the corneal endothelial -like cells were viable and showed polygonal morphology with expression and native-like localization of ZO-1, Na^+^/K^+^-ATPase and CD166. However, mesenchymal-like cells were observed in certain areas of the cultures, spreading beneath the corneal endothelial-like cell layer.

**Conclusions:**

Our results demonstrate the successful printing of human pluripotent stem cell derived corneal endothelial cells using covalently crosslinked hyaluronic acid bioink. This approach not only holds promise for a corneal endothelium transplants but also presents potential applications in the broader mission of bioprinting the full-thickness human cornea.

**Supplementary Information:**

The online version contains supplementary material available at 10.1186/s13287-024-03672-w.

## Background

Corneal transplantation from cadaveric donors is effectively used for the treatment of corneal blindness. Unfortunately, the increase in transplantation activity aggravates the global donor tissue shortage and it has been estimated that there is only one donor cornea available for every seventy patients in need and 12.7 million people require a corneal transplantation worldwide [1]. Importantly, the most common reason for the corneal transplantation is problems related to the impairment of corneal endothelium [2]—a monolayer of cells which main function is to transport water out of the stroma to maintain corneal clarity [3, 4]. These highly polarized and flat human corneal endothelial cells (hCEnC) with diameter of approximately 20 µm [5] have tight hexagonal apical surface, but their basal surface is irregular, as they lie on an amorphous collagenous membrane called Descemet membrane (DM) [4]. Relative hydrated cornea is sustained by “Pump-Leak” mechanism, where active transport properties including sodium–potassium pump (Na^+^/K^+^ ATPase) represent the “Pump” and the pressure caused by the swelling cornea presents the “leak”. The barrier properties of tight junction proteins such as Zona Occludens-1 (ZO-1) regulates this mechanism. [6, 7] After embryonic development, hCEnCs are arrested at G1 phase, thus they are unable to divide, and cornea endothelium lacks regenerative capacity through cell division [6]. Recent advances in surgical technologies including endothelial keratoplasty (such as DSEK and DMEK) allow for the selective replacement of diseased cornea endothelium with that of a donor [8]. For the worldwide shortage of donor corneas, advanced tissue engineering and manufacturing technologies including 3D bioprinting could offer great opportunities in future to produce native-like 3D corneal structures using functional bioinks and scalable human stem cells.

Corneal tissue, in general, is an attractive target for 3D bioprinting technology since it has relatively low thickness and lack of vascularization, which have been major restrains in 3D printing technology in many other applications including bone, heart, liver or skin tissue [9, 10]. In any clinical 3D bioprinting application, a suitable bioink is required in addition to a functional and scalable cell source, both ideally based on xeno-free components [10]. For the summary of the current state-of-the-art in 3D bioprinting of cornea, recent review article by Jia and co-authors is available [11]. Human cornea stroma mimicking structures have been 3D bioprinted with extrusion-based printing [12], laser-assisted 3D bioprinting [10] as well as stereolithography [13]. Towards this end, we have previously investigated the possibility to 3D bioprint human cornea mimicking structures with human stem cell produced epithelial and stromal layers [10], as well as developed a novel hyaluronic acid (HA)-based dopamine containing bioink using hydrazone crosslinking chemistry for the 3D bioprinting of corneal stroma equivalents with high tissue integration [14]. Due to the challenging non-proliferative nature of hCEnCs, there is to the best of our knowledge only one published study focusing on bioprinting of CEnCs, using RNase 5 vector-transfected human primary CEnCs and gelatin based bioink [15].

**Fig. 1:**
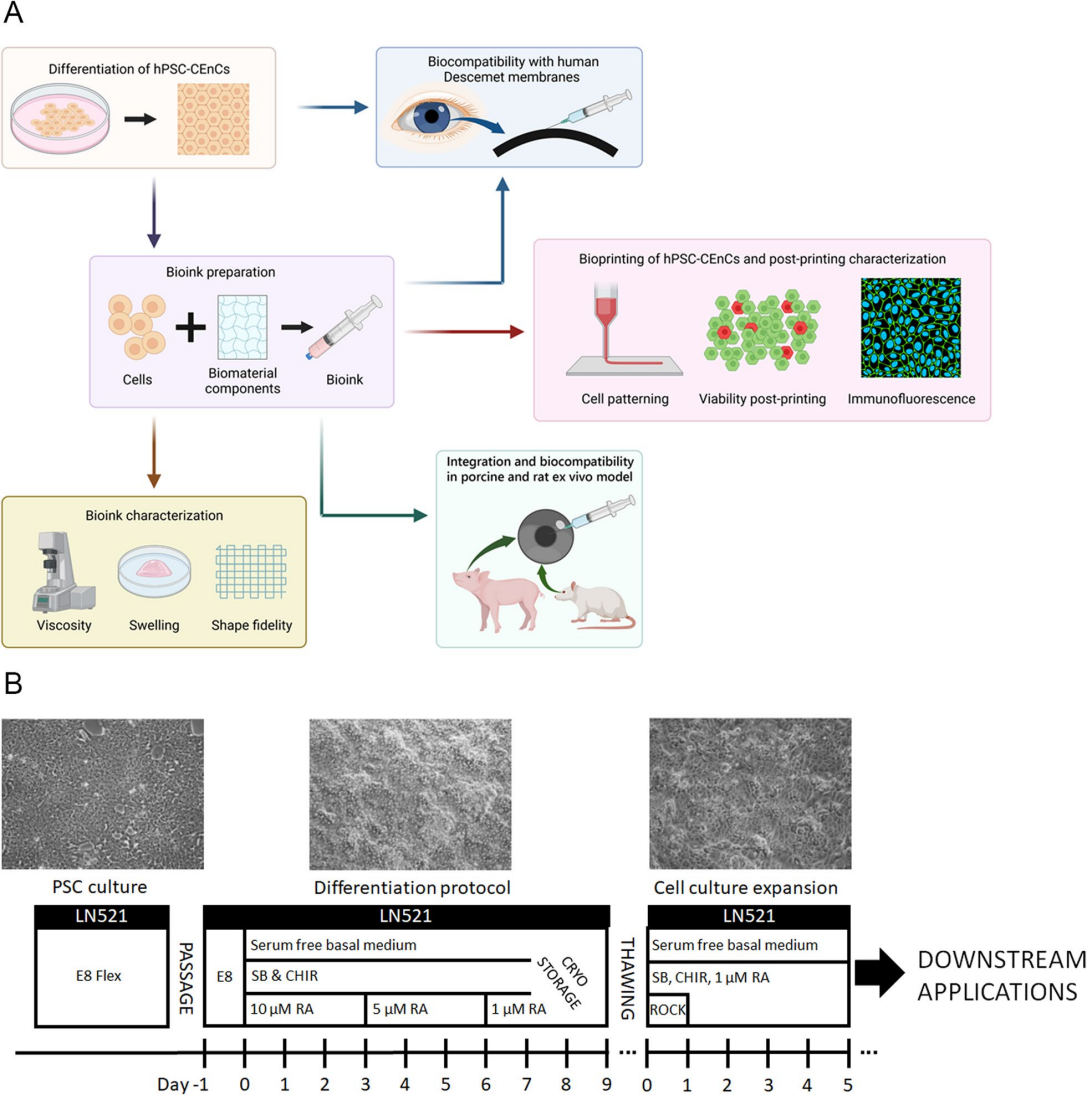
**A** Schematic illustration of the workflow of this study. Created with Biorender.com. **B** Schematic presentation of the hPSC-CEnC differentiation for bioprinting. *LN521* Human recombinant laminin 521, *FIBRIN* Fibrin membrane, *E8 Flex* Essential 8 Flex Medium, *SB* Activin/BMP/TGF-β pathway inhibitor SB431542, *CHIR* GSK-3 inhibitor and WNT pathway activator CHIR99021, *RA* Retinoic acid, *ROCK* ROCK inhibitor Y27632

In this study, our aim is to formulate a biocompatible and functional bioink specifically for hCEnCs, ensuring optimal printability, viability, preservation of the hCEnCs phenotype and tissue integration throughout the bioprinting process. (Fig. 1A). Importantly, we hypothesized that the functional bioink should enable the bioprinting of microscale structure of corneal endothelium, provide natural extracellular matrix environment for cells and support the monolayer formation and maturation of the corneal endothelium after the bioprinting. Furthermore, to tackle the current donor tissue scarcity, the de novo generation of hCEnCs from human pluripotent stem cells (hPSC) is appealing solution [16–25]. By combining hPSC-CEnC-like cells produced with previously established production method [16] and optimized bioink, we aim to provide technological solutions for the crucial endothelial layer in the mission of 3D bioprinting of the full-thickness human cornea.

## Methods

### Preparation of the bioink

Here, we used our previously developed (HA)-based hydrazone crosslinked bioink [14] with modifications optimal for the bioprinting of cornea endothelium. The synthesis and characterization of crosslinking components, that is carbohydrazide (CDH) conjugated dopamine modified HA (HA-DA-CDH) and aldehyde conjugated HA (HA-ALD), has been previously described [26, 27]. In this study, the crosslinking components were dissolved into 1X Dulbecco’s Phosphate Buffered Saline (DPBS) into concentration of 6 mgml^−1^. Unmodified sodium hyaluronate (Novamatrix, Norway) with a concentration of 1% (w/v) was used as a primary rheological modifier. Human collagen IV (Col IV) from human placenta (Sigma–Aldrich St. Louis, MO, USA) and human recombinant laminin 521 (LN521™, Biolamina, Sweden) were used as additional rheological modifiers in the bioink and to improve the cytocompatibility and functionality for hPSC-CEnCs. Col IV was dissolved into dilute acetic acid (Merck, Germany) to obtain a solution of 2.5 mgml^−1^. Just before mixing the bioink, the Col IV was neutralized into pH 7 with 1 M NaOH and 10 × DPBS (Carl Roth, Karlsruhe, Germany). LN521™ was used in a concentration of 0.1 mgml^−1^. The mixing of the bioink components has been previously described [14]. The bioink was crosslinked at room temperature for 90 min before 3D bioprinting.

### Shear thinning

Shear-thinning properties of the bioinks with and without cells were analyzed with viscosity measurements to determine if cells affect the viscosity. Viscosity was measured with HR-2 Discovery hybrid rheometer (TA Instruments, DE, USA) under continuous flow using 20 mm parallel plate geometry, 1 mm gap and shear rate ranging from 0.01 to 10 1 s^−1^. Bioinks were prepared as previously described [14] and pre-crosslinked in the syringes for 100 min. The cell density of the cell-laden bioink was 6 million cellsml^−1^. After pre-crosslinking, 400 µl bioink samples were injected onto the rheometer bottom plate for measuring. The measurements were carried out within 80 min after starting the first measurement, and five replicates per bioink were measured (n = 5).

### 3D bioprinting setup

Extrusion-based 3D bioprinter 3D-Bioplotter® Manufacturer Series by EnvisionTEC (Gladbeck, Germany) was used. Before printing, the bioink was placed in a 30 cc Nordson EFD syringe barrel (OH, USA). Small 32G blunt needles with 100 µm inner diameter were chosen for printing (0.50 inches, CellInk, Sweden, Gothenburg). All the 3D bioprinting experiments were carried out at room temperature. 3D models in stl format were prepared with Perfactory RP Software (EnvisionTEC, Gladbeck, Germany) and printing patterns and parameters were adjusted in Visual Machine (EnvisionTEC, Gladbeck, Germany). Slice interval of 70% of the nozzle inner diameter was used for all printed structures, and the distance of the first layer was set at 0.07 mm.

Two patterns were used for printing hPSC-CEnCs. First, line patterns were used to study the patternability of hPSC-CEnCs. Here, two layers of aligned lines with 1.3 mm distance between lines were printed using printing parameters of 0.4 bar and 13 mms^-1^ speed. To further study the 3D bioprinting of hPSC-CEnCs, two layers of hPSC-CEnC containing bioink were printed onto fibrin membrane in a form of a low cylinder with a diameter of 10 mm. Line distance of 0.30 mm was used, and the adjacent layers were printed at 90° angle to the previous layer. Printing parameters of 0.6 bar and speed 10 mms^−1^ were chosen. For these studies, hPSC-CEnCs density in the bioink was 10 million cells ml^-1^. The fibrin membrane used as printing substrate was prepared from a commercially available two-component surgical fibrin sealant (Tisseel, Baxter, IL, USA). Before use, the fibrinogen component was diluted 1:1 with 2.9% NaCl (VWR), 2.6 mM CaCl2 (Sigma-Aldrich) and the thrombin component 1:166 with 1.1% NaCl; 1 mM CaCl2. For fibrin formation, the fibrinogen and thrombin components were combined, pressed onto a bioassay dish with the help of two sterilized parafilm strips to form a membrane, and left to jellify for 30 minutes RT.

### Shape fidelity

The uniformity and shape fidelity of deposited HA-based bioinks was investigated to evaluate the printability. To explore the shape fidelity of the HA-based bioink, the filament thickness and pore factor (Pr) of printed grids were determined as a function of time. HA-based bioink was prepared as described above and allowed to pre-crosslink for 90 min. Grids of two printed layers with dimensions of 20 mm × 20 mm were 3D bioprinted onto 35 mm dishes. Distance between lines of 2.50 mm were used and filaments in alternative layers were printed perpendicularly. Printing pressure of 0.4 bar and speed of 17.0 mms^−1^ was used. The samples were imaged with a high-definition CCD-camera attached to the dispenser head mount immediately after printing and after 7 days submerged in PBS at + 37 °C. The thickness of the printed hydrogel filaments and pore geometry was quantified with Image J image processing and analysis software. For each printed sample (n = 6) and time point, the filament thickness of 2 adjacent layers was evaluated from 9 different filaments. Six randomly selected pores in each printed sample (n = 3) were included in image analysis. Pr was counted according to the following equation, where L means perimeter of the pore and A the pore area.

$$Pr=\frac{{L}^{2}}{16A}$$

The statistical data analysis of shape fidelity measurements was determined with non-parametric Mann–Whitney U test (IBM SPSS Statistics software). P-values ⩽ 0.05 were considered statistically significant.

### Swelling behavior and degradation of the bioink

To study the degradation and swelling characteristics of the bioink in in vitro setting, three parallel bioink samples (n = 3) were subjected to neutral pH conditions in PBS. 200 µl samples of bioink was injected onto 35 mm diameter dishes. The samples were allowed to stabilize for 90 min and subsequently submerged into PBS. After 30 min, the PBS was removed, and the initial weight of the samples was recorded. Thereafter, the bioink samples were incubated in PBS at + 37 °C. 1, 3, 7 and 10 days of post-printing, the PBS was removed thoroughly from the samples, and weight of the remaining samples was recorded. The swelling behavior of the HA-based bioink was analyzed by calculating the remaining weight percentages of the samples at each timepoint according to the following formula:

$$\mathrm{Remaining\, weight \% }=\frac{\mathrm{Measure\, weight}}{\mathrm{Initial\, Weight}}\times 100$$

### Differentiation and cryopreservation of hPSCs-CEnC-like cells

The previously established and characterized hPSC lines were used in this study including, human embryonic stem cell (hESC) line Regea08/017 [28] and human induced pluripotent stem cell (hiPSC) line WT001.TAU.bB2 [16]. The hPSC-CEnCs used for the experiments were produced mainly with the hESC line unless indicated differently. CEnC-like cells were differentiated from hPSCs based on our previously established protocol [16] with small retinoic acid (RA) concentration modifications (Fig. 1B). Briefly, hPSC differentiation was carried out on laminin-521 coated 6 or 12 well-plates (Corning) at 20,000–40,000 cells cm^−2^. The hPSCs were cultured in E8 medium for 24 h. On day 0 of differentiation, the medium was switched to serum free basal medium consisting of KnockOut Dulbecco’s Modified Eagle Medium (KO-DMEM), 15% Knock-out serum replacement (KO-SR), 2 mM GlutaMax-I, 0.1 mM 2-mercaptoethanol, 50 Uml^−1^ Penicillin/Streptomycin (all from Thermo Fisher), 1% Non-essential Amino Acids (Thermo Fisher, Sigma-Aldrich) supplemented with 10 µM TGF-β inhibitor SB431542 (SB; Stemcell), 4 µM GSK3 inhibitor/WNT pathway activator CHIR99021 (CHIR; Stemcell) and 10 µM RA (Sigma-Aldrich). RA concentration was lowered to 5 µM during the days 3–6 and then lowered further to 1 µM. During the days 7–9, CEnC-like cells had formed, and they were cryopreserved. For cryopreservation, cells were harvested using TrypLE Select (Thermo Fisher) dissociation enzyme and incubated for 3–6 min at + 37 °C. Subsequently, the CEnC-like cells were detached gently by trituration, filtered through 40 µm Cell Strainer (Thermo Fisher) and centrifuged for 5 min at 300 g. The cells were resuspended in cryomedium consisting of basal medium with 40% KO-SR and 10% dimethyl sulfoxide (DMSO, Sigma) in 2 ml cryogenic storage vial (Sarstedt).

After cryopreservation, the cells were thawed and passaged on LN521™ coated 6 or 12 well-plates at 200,000–300,000 cellscm^−2^ in basal medium supplemented with 10 µM SB, 4 µM CHIR, 1 µM RA and 10 µM ROCK inhibitor Y27632 (ROCKi, Stemcell). After 24 h, ROCKi was removed from the medium. Cells were cultured for 5 days and then harvested for bioprinting. Contrast light microscope Nikon Eclipse TE2000-S with a DS-Fi1 camera (Nikon Corp. Tokyo, Japan) was used to capture images of the cell morphology. The data in this study was produced from 8 individual batches of hESC-CEnCs and 2 individual batches of hiPSC-CEnCs**.**

### Flow cytometry

Flow cytometry (FC) was used to quantify the expression of CD166 10 days from the beginning of the differentiation of the hPSC-CEnCs (WT001.TAU.bB2 hiPSC-line). For FC staining, cells were detached with TrypLE Select and Defined Trypsin Inhibitor (DTI, Thermo Fisher), centrifuged 5 min at 1000 rpm, resuspended in medium and counted. 1 × 10^5^ cells/100 µL per sample were divided to 5 ml sample tubes and the cells were washed twice by centrifuging 2 min at 1900 rpm in FC wash buffer containing 0.5% BSA and 1 mM ethylenediamine-tetraacetic acid (EDTA; Gibco, Thermo Fisher Scientific) in DPBS. Next, the samples were incubated with PE-conjugated mouse anti-human CD166 primary antibody (#559,263, BD Biosciences, NJ, USA) for 20 min in the dark on ice. BD Pharmingen™ PE Mouse IgG1, κ Isotype Control (#555,749, BD Biosciences) was used along with unstained control. Samples were washed twice with the FC washing buffer and immediately analyzed with the CytoFLEX S Flow Cytometer (Beckman Coulter Life Sciences, IN, USA) collecting 10 000 events of the primarily gated population of interest. The collected data were further analyzed with the CytExpert Software. In CytExpert, the negative control was used to gate the population of interest containing the cells. After excluding doublets from the analysis, the negative vs. positive gates were set with histograms using 0.5% marginal. Finally, the established gates were copied to each sample of the experiment.

### Rat cornea organ culture for the biocompatibility and integration test of the bioink

Corneal tissues (n = 6) were obtained from the eyeballs extracted from three euthanized Sprague Dawley rats. Briefly, the ocular surface was cleaned using saline solution before excision. For excision, the rat eyes were held on the bulbar conjunctiva using a toothed forceps and gently pulled outward. Thereafter, fornix conjunctiva was first excised all around the eye using sterile curved ophthalmic scissors with serrated tips. Later, intraocular muscles and optic nerve were gently excised to extract the eyeball. During the procedure, care was taken to avoid bleeding to prevent additional corneal contamination. Eyeballs were immediately placed in the basal medium and were transferred to a sterile hood. Further, the eyeballs were placed in the basal medium containing 400 units ml^−1^ of penicillin–streptomycin and were incubated for 30 min at 37 °C and 5% CO_2_.

For corneal extraction, eyeballs were placed in a petri dish containing medium to prevent drying. Using a stereo zoom microscope, a 21-gauge needle was used to pierce the rat retinal area, after which the peripheral cornea was nicked using a scalpel blade no. 15. Later, curved Vannas scissors with sharp tips were used to cut the corneal tissue, which were then transferred to a new petri dish with the medium. Rat corneas were treated with TrypLe Select over night to remove native corneal endothelium. Corneas were moved into 96-well plate wells with cut bottom part of sterile 1.5 ml Eppendorf tube to give the well U-shaped bottom which helps the cornea to stay endothelial side up. Next, hPSC-CEnCs in bioink were injected on three corneas (n = 3) and hPSC-CEnCs without bioink were seeded on the other three corneas (n = 3) at 300 000 cells cm^−2^ according to 96-well plate surface area. After that, hPSC-CEnCs were cultured 5 days in hPSC-CEnC differentiation medium with 1 µM RA and first 24 h also with 10 µM ROCKi at + 37 °C in 5% CO_2_. For analyses, two corneas with bioink and cells, and two corneas seeded with cells were fixed for flat mount immunofluorescence staining. In addition, one cornea from each sample type were fixed for cryosections and staining.

### Porcine cornea organ culture for biocompatibility and integration test of the bioink

The corneal organ culture using excised porcine corneas was performed as previously described [10, 29] with minor modifications. Briefly, the excess tissue was stripped from the whole fresh porcine eyes after which they were disinfected with 2% povidone iodine (Betadine®, Leiras, Helsinki, Finland). Corneas were then dissected from the eyes in aseptic conditions and further disinfected in 1% povidone iodine. The corneas were put overnight in Advanced DMEM supplemented with 1% GlutaMAX™, 1% Penicillin–Streptomycin and 0.25 μgml^−1^ amphotericin B (Thermo Fisher Scientific) at + 37 °C in 5% CO_2_ to prevent bacterial contamination and then overnight in trypsin–EDTA (0.25%) (Thermo Fisher) to dissociate porcine primary CEnCs and corneal epithelial cells (CEpC) from the cornea. Remaining primary CEnCs and CEpCs were removed gently with blunt forceps without detaching the DM. Porcine corneas (n = 2) were put on 12 well plates DM side up and the bioink comprised with hPSC-CEnCs was injected to cover the whole DM. Corneas were cultured in the hPSC-CEnC differentiation medium with 1 µM RA and first 24 h with 10 µM ROCKi at + 37 °C in 5% CO_2_ for 10 days before freezing for cryosections.

### Human DM from donor cornea for biocompatibility and integration test of the bioink

The human donor corneas (Regea tissue bank, Tampere University, Finland) not suitable for clinical use (n = 6) were stored in CorneaMax storage medium (Eurobio Scientific, France). To separate DMs from the corneal stroma, each cornea was handled in the following manner. Storage medium was injected using a 27-gauge needle into the corneal stroma underneath the DM to create a liquid filled bubble. The injection site was enlarged using the same needle to deflate the bubble and to prevent the membrane from bursting during cutting. Then, with an 8 mm biopsy punch, the DM was cut off and transferred to the DPBS solution. Long storage time (minimum of 3 months) and rinsing with DPBS had denuded the DM from primary CEnCs. DMs were moved to 24 well plate and they were pinned on the bottom of the well with CellCrown™ 24NX (Scaffdex, Tampere, Finland) or with small metal ring to prevent floating. The bioink comprised with hPSC-CEnCs was injected on the DM. As a control, hPSC-CEnCs were seeded without bioink on the DM with density of 150 000 cellscm^−2^. Cells on DM were cultured in the hPSC-CEnC differentiation medium with 1 µM RA and first 24 h with 10 µM ROCKi at + 37 °C in 5% CO_2_ for 6 days before fixing for immunofluorescence staining.

### Bioink cytocompatibility with bioprinted hPSC-CEnCs

The cytocompatibility of the bioink was evaluated using LIVE/DEAD® viability/cytotoxicity kit with bioprinted hPSC-CEnCs on fibrin membrane. Briefly, the cells were washed with DPBS and incubated with Live/Dead staining solution containing 2 µM Calcein AM and 1 µM Ethidium homodimer diluted in DPBS in room temperature for 30 min. The cells were washed and imaged using a fluorescence microscope (Olympus IX51; Olympus, Tokyo, Japan) or confocal microscope (Zeiss LSM 800, Carl Zeiss AG, Germany). The percentage of dead cells was manually calculated from the images.

### Immunocytochemistry of the hPSC-CEnCs

After differentiation, the phenotype of hPSC-CEnCs used in the experiments were analyzed with immunocytochemistry. For that, the cells were fixed with 4% paraformaldehyde (PFA, Sigma-Aldrich) for 15 min. Next, the cells were permeabilized for 10 min with 0.1% Triton X-100 (Sigma-Aldrich) followed by blocking with 3% bovine serum albumin (BSA) for 1 h. Then the cells were first incubated with 1:400 ZO-1 (#61–7300, Thermo Fisher) 1:200 Na^+^/K^+^-ATPase (#ab7671, Abcam, #55,187–1-AP, Proteintech, IL, USA), 1:400 CD166 (#559,260, BD Biosciences), STEM121 1:100 (#Y40410, Takara Bio Inc., Japan), 1:400 Ki67 (#AB9260, Millipore,F MA, USA), 1:400 Keratocan (sc-33243, Santa-Cruz, Dallas, TX, USA), 1:400 PAX6 (HPA030775, Sigma-Aldrich) primary antibodies and 1:200 phalloidin Alexa Fluor 647 (Thermo Fisher) overnight at 4 °C. The cells were next treated with 1:800 Donkey anti-Rabbit IgG Secondary Antibody, Alexa Fluor 488, 1:800 Donkey anti-Mouse IgG Secondary Antibody, Alexa Fluor 568 (both from Thermo Fisher) and 1:800 Donkey anti-Goat IgG Secondary Antibody, Alexa Fluor 647 (Abcam) according to the host of the primary antibody for 1 h at room temperature. The nuclei were counterstained with 1:1000 Hoechst 33,342 (Thermo Fisher) with secondary antibody incubation or with 4’,6-diamidine-2’-phenylindole dihydrochloride in mounting medium (DAPI; Vector Laboratories, Peterborough, UK).

The flat mount technique was employed for rat corneas. Briefly, three cuts were made with scalpel blade no. 10 around the cornea approximately 1/3 of the diameter of the cornea before mounting. Vectashield Antifade Mounting Medium (Vector Laboratories) was used for mounting. The images of stained cells were captured using a fluorescence microscope (Olympus IX51; Olympus, Tokyo, Japan) or confocal microscope (Zeiss LSM 800, Carl Zeiss AG, Germany) and prepared using image editing software (Adobe Photoshop CC 2021; Adobe Systems) or Zen 3.0 (Blue edition) (Carl Zeiss AG).

### Immunohistochemistry of the cells on fibrin membrane, ex vivo rat and porcine corneas

The hPSC-CEnCs bioprinted on fibrin membrane, injected with bioink or seeded without bioink on ex vivo rat and porcine corneas were fixed with 4% PFA for 3 h and put into 30% sucrose overnight at + 4 °C. Then, the samples were put into Tissue-Tek® O.C.T. Compound (Sakura Finetek, USA) and kept overnight at + 4 °C. For snap freezing, samples were put vertically in cryomolds filled with O.C.T. Molds were then submerged into isopentane in decanter glass which was surrounded by liquid nitrogen. Frozen samples were stored at -80 °C. The frozen sample blocks were cut into 8 µm thick sections with cryostat (MEV + , SLEE medical GmbH, Germany). The sections on glass slides were stained for the evaluation of the tissue integration with hematoxylin and eosin (H&E) using standard protocols. Next, the sections were permeabilized with 0.1% Triton X-100. Sections were treated with blocking buffer consisting of 5% BSA for 1 h in moisture chamber at room temperature. Then the sections were first incubated with 1:200 ZO-1, 1:100 Na^+^/K^+^-ATPase 1:200 CD166 and anti-human cytoplasm STEM121 1:80 (for porcine and rat ex vivo sections) primary antibodies in blocking buffer in moisture chamber overnight at 4 °C. After 20 min DPBS wash, the sections were incubated in 1:400 Donkey anti-Rabbit IgG Secondary Antibody, Alexa Fluor 488, 1:400 Donkey anti-Mouse IgG Secondary Antibody, Alexa Fluor 568 and 1:500 Hoechst 33,342 in blocking buffer for 1 h in moisture chamber at + 37 °C. Sections were washed for 20 min in DPBS and mounted with Prolong Gold Antifade Mountant (Thermo Fisher). The images of mounted cells/tissue were captured using using a fluorescence microscope (Olympus IX51; Olympus, Tokyo, Japan) or confocal microscope (Zeiss LSM 800, Carl Zeiss AG, Germany) and prepared using image editing software (Adobe Photoshop CC 2021; Adobe Systems) and Zen 3.0 (Blue edition) (Carl Zeiss AG).

### Transendothelial electrical resistance measurement

Transendothelial electrical resistance (TEER) of the hPSC-CEnCs was measured with Millicell electrical resistance system volt-ohm meter (Merck Millipore, Darmstad, Germany). TEER values were measured from hPSC-CEnCs cultured for 6 days on 24 well plate hanging cell culture inserts with pore size 1.0 µm (Sarstedt, Germany). TEER values were obtained from four different layouts: bioink with cells (n = 4), seeded cells without bioink (n = 4), blank empty insert (n = 4) and bioink without cells (n = 3). All samples were treated with identical medium changes. Measured TEER values (Ωcm^2^) were multiplied by the surface area of the insert (0.3 cm^2^). Average of the values with each layout were calculated and the TEER values of blank empty insert was subtracted from the result. The statistical data analysis of TEER measurements was determined with non-parametric Mann–Whitney U test (IBM SPSS Statistics software). A *P*-value ⩽ 0.05 was considered statistically significant.

### Ca^2+^ imaging and cell size analysis

For Ca^2+^ imaging, the hPSC-CEnCs injected with bioink (n = 2) or seeded (100 000 cells cm^−2^) without bioink (n = 2) were cultured for 6 days on sterile 13 mm diameter glass coverslips (VWR, PA, USA). The ATP-induced Ca2 + responses was measured as described in [29–31]. Briefly, the cells were loaded with permeable Ca^2+^ sensitive fluorescent dye fluo-4-acetoxymethyl ester (1 mM, fluo-4 AM; Thermo Fischer Scientific) diluted in Elliot buffer solution (pH 7.4, osm 330 mOsm) for 30 min RT. After loading, the cells were washed with Elliot buffer for 10 min RT. During imaging, the cells were perfused with pre-warmed (approx. 37 °C) Elliot solution alone or Elliot containing 100 µM ATP (Sigma-Aldrich). Nikon Eclipse FN1 upright fluorescence microscope with water immersion 25 × objective (NA = 1.10) was used. The cells were imaged for 10 min which included 5 min of baseline imaging, 2 min of ATP stimulus and 3 min of additional imaging. All steps in the Ca^2+^ imaging were performed protected from light. Data analysis was performed with ImageJ and MATLAB (R2021a) as described previously [31]. Responsive cells were calculated from three randomly selected region of interests (ROI, 200 × 200 pixels) from each replicate to reach total of 6 analyzed ROIs per sample type.

In addition, cell numbers from the ROIs during the Ca^2+^ imaging were manually counted and used to compare the representative cell size of bioink and seeded samples. For additional quantification, cells injected with bioink and seeded without bioink were cultured on cell culture plastics for 6 days and imaged with Contrast light microscope Nikon Eclipse TE2000-S with a DS-Fi1 camera (Nikon Corp. Tokyo, Japan). For quantification, 14–30 ROIs (500 × 500 pixels) were chosen from the CEnC-like cell areas and the cells were counted manually. Furthermore, the average cell sizes in the cell cultures were measured using NucleoCounter® NC-200™ (Chemometec, Allerod, Denmark). This involved 27 individual hPSC-CEnCs in bioink and 7 seeded hPSC-CEnCs (both from Regea08/017 line), as well as 190 hPSC-CEnCs in bioink and 89 seeded hPSC-CEnCs (both from the WT001.TAU.bB2 line). All statistics were performed with Mann–Whitney U with GraphPad Prism (version 5.02) to compare statistical significances. A *p*-value of ⩽ 0.05 was considered statistically significant.

### RNA extraction and real-time qPCR

Total RNA was extracted from hPSC-CEnCs injected with bioink and seeded without bioink on cell culture plastics (d6) using TRI reagent (Sigma-Aldrich). RNA concentration of each sample was determined using NanoDrop-1000 spectrophotometer (NanoDrop Technologies, Wilmington, DE, USA). RNA was purified from endogenous DNA using Dnase I (Thermo Fisher Scientific). From each RNA sample 400 ng were used to synthesize cDNA using the High-Capacity cDNA RT kit (Applied Biosystems, Foster City, CA, USA). The resulting cDNA samples were analyzed with qPCR using sequence-specific TaqMan Gene Expression Assays (Thermo Fisher) for ATCAM (CD166, Hs00977641_m1). All samples were run as triplicate reactions with the QuantStudio 12 K Flex Real-Time PCR System (Applied Biosystems). Results were analyzed with the QuantStudio 12 K Flex Software (Applied Biosystems) and Microsoft Excel. Based on the cycle threshold (C_T_) values given by the software, the relative quantification of each gene was calculated by applying the -2^ΔΔCt^ method [33]. Results were normalized to GAPDH (Hs99999905_m1), with the hPSC-CEnCs injected with bioink as the calibrator to determine the relative quantities of gene expression in each sample.

## Results

### Developed bioink demonstrates good printability and shape fidelity

**Fig. 2:**
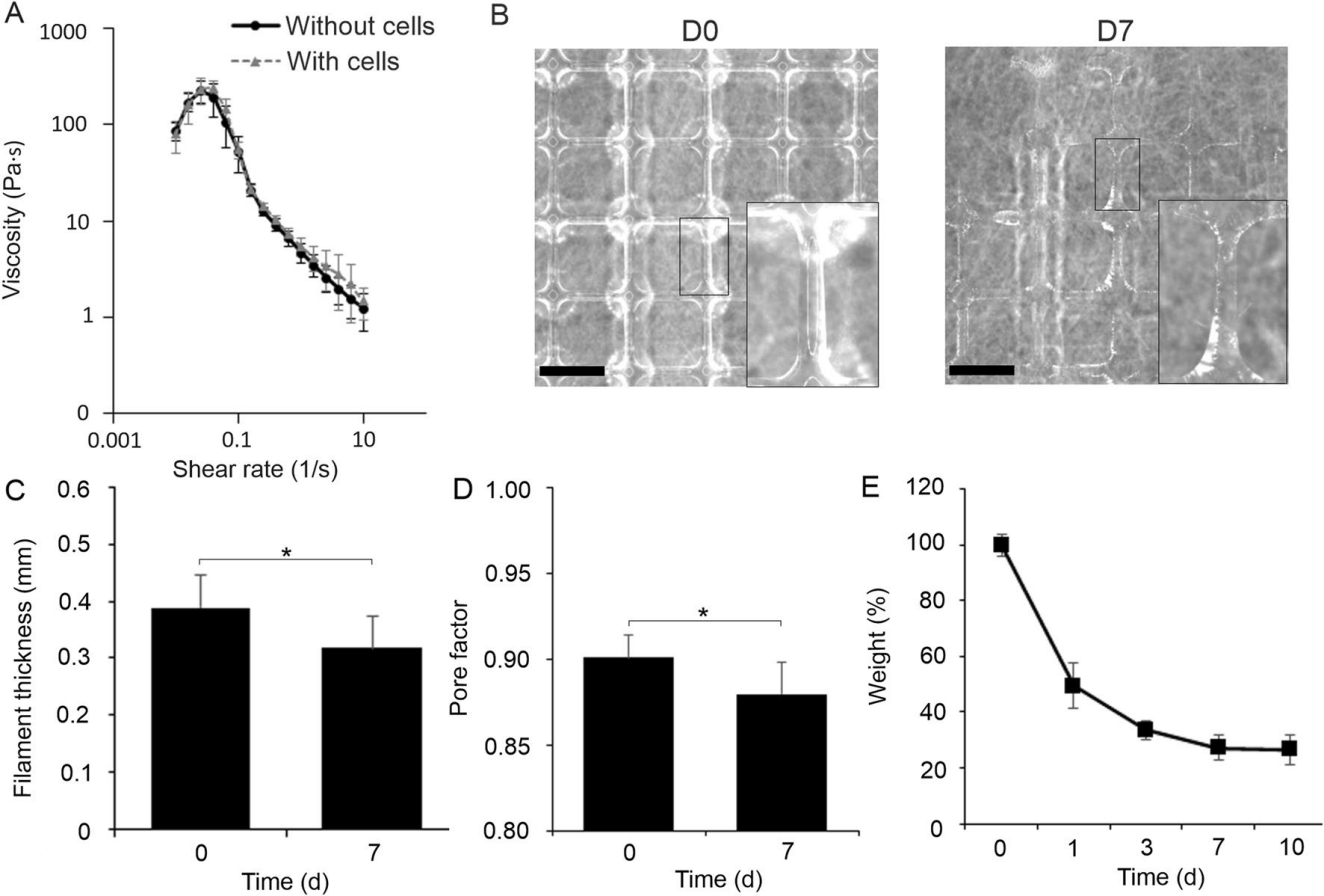
The bioink shows good printability and shape fidelity. **A** Viscosity measurements under continuous flow without and with hPSC-CEnCs differentiated from the used hESC line. Five replicates per bioink were measured (n = 5). **B** Images of printed lattices after the printing and 7 days in culture. Scale bars 2 mm. **C** Filament thickness (n = 6 printed structures) and **D** pore factors were analyzed (n = 3 printed structures) from the printed structures at day 0 and 7 to explore shape fidelity after bioprinting (* = p < 0.05). **E** Degradation and swelling behavior of the bioink upon time (n = 3 bioink samples)

Shear-thinning properties of the bioinks with and without cells were determined with viscosity measurements under continuous flow. Both bioinks demonstrated shear-thinning properties when shear rate increased (Fig. 2A). However, the range of viscosity values was low for both bioinks. The viscosity of the bioink with cells was 241 ± 42 Pa·s and the bioink without cells 226 ± 58 Pa·s. Thus, only slight increase was seen in the viscosity of the bioink with cells and the difference was not significant. To investigate the filament formation and shape fidelity of the bioink, two-layered grids were printed with bioink without cells (Fig. 2B). The bioink showed good printability with 32G nozzles and demonstrated formation of continuous filaments and grid structures (Fig. 2B) in the printing process. The filament thickness was 388 ± 65 µm immediately after printing (Fig. 2C). After 7 days in culture, a clear visible grid structure was seen. Some breakage of the filaments could be detected, but majority of the filaments and pores were intact after the 7 days culture period. Slight decrease in filament thickness was seen with filament thickness of 318 ± 58 µm at day 7 (p < 0.001), indicating loss of small amount of material during the culture period. Immediately after printing, Pr value of 0.90 ± 0.01 was measured. Small decrease in Pr value down to 0.88 ± 0.02 was also seen at day 7 after printing (Fig. 2D) (p < 0.001). Individual datapoints for filament thickness and Pr are shown in Additional file 10: Fig. S1. A representative original image used for image analysis of bioink shape fidelity is illustrated in Additional file 11: Fig. S2. The stability and swelling behavior of the bioink was also explored by immersing the bioink without cells in PBS under neutral conditions and calculating the change in weight % over 10 days (Fig. 2E). The weight of the bioink decreased significantly until day 7. Thereafter, the weight loss levelled out and no further weight loss was recorded. At day 10, only 26 ± 5% of the original weight of the bioink was measured.

### Biocompatibility and integration of the bioink with hPSC-CEnCs to rat and porcine cornea and human DM

**Fig. 3:**
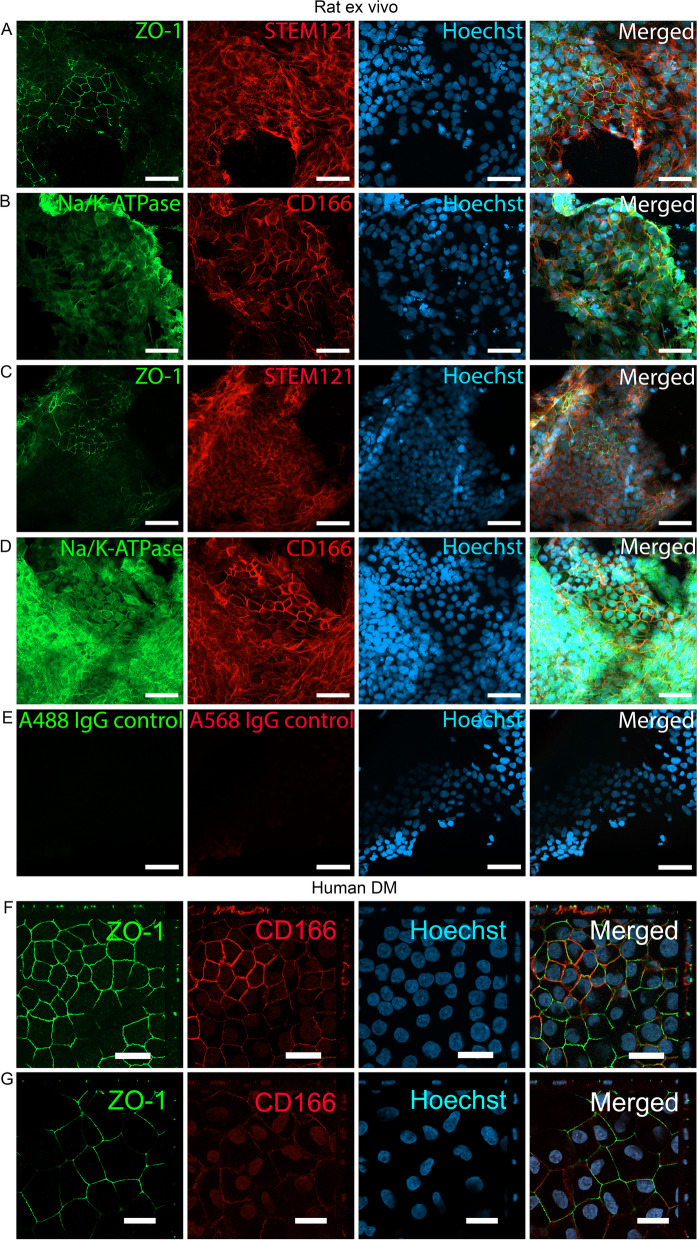
The bioink shows good biocompatibility and integration on rat ex vivo cornea (n = 2 rats including total of 4 corneas) and human DM (n = 6 donors). hPSC-CEnCs differentiated from the used hESC line. **A**, **B** Representative flat mount immunofluorescence stainings of hPSC-CEnCs in bioink injected on rat ex vivo cornea (n = 2) after 5 days of injection and **C**, **D** hPSC-CEnCs seeded without bioink on rat ex vivo cornea (n = 2) after 5 days of seeding. **E** Representative IgG controls of hPSC-CEnCs seeded without bioink on rat ex vivo cornea. **F** Representative images of hPSC-CEnCs in bioink injected on human DM (n = 3) and G) hPSC-CEnCs seeded without bioink on human DM (n = 3) after 6 days of injection/seeding. In **A**, **C**) ZO-1 (green) STEM121 (red) and Hoechst (cyan); **B**, **D**) Na^+^/K^+^-ATPase (green) CD166 (red) and Hoechst (cyan); E) IgG control for Alexa Fluor 488 (green), IgG control for Alexa Fluor 568 (red) and Hoechst (cyan); **F–G** ZO-1 (green) CD166 (red) and Hoechst (cyan). Scale bars 50 µm (**A**–**E**), 20 µm (**F**–**G**)

The phenotype of the hPSC-CEnCs was confirmed before used for experiments (Additional file 12: Fig. S3A–C). Also, some unidentified cell populations appeared in the hPSC-CEnCs cultures before experiments (Additional file 12: Fig. S3D–F) and part of those cells were positive for keratocan or PAX6 markers. To analyze the biocompatibility and integration of the bioink with hPSC-CEnCs, ex vivo rat cornea model (6 corneas from 3 rats) was used to demonstrate cell attachment to the native DM with the bioink (Fig. 3A–B, Additional file 1: Fig. S4A–B and Additional file 2: Fig. S5A). Immunofluorescence marker STEM121 was used to verify the human origin of the cells on the DM of the ex vivo rat corneas after 5 days of bioink injection or seeding without bioink (Fig. 3A, C). STEM121 was expressed only on rat DM where the hPSC-CEnCs containing bioink was injected or seeded without bioink but not in the native rat stroma (Additional file 1: Fig. S4A, C). The ZO-1, Na^+^/K^+^-ATPase and CD166 staining demonstrated typical CEnC characteristics on rat DM with ZO-1 expressed on tight junction of the cells, and Na^+^/K^+^-ATPase and CD166 expression in the lateral cell membrane (Fig. 3A–B, Additional file 1: Fig. S4A–B). The results resembled the control where hPSC-CEnCs were seeded without bioink (Fig. 3C–D, Additional file 1: Fig. S4C–D). Furthermore, attachment of the hPSC-CEnCs injected with bioink on porcine DM was seen in the porcine cornea ex vivo tests (2 corneas from 2 pigs) (Additional file 1: Fig. S4F–G and Additional file 2: Fig. S5B).

The biocompatibility and integration of the bioink with hPSC-CEnCs was tested on human DM (n = 3 donors) (Fig. 3F, Additional file 3: Fig. S6A, C and Additional file 4: Fig. S7A–B) using hPSC-CEnCs seeded without bioink on human DM (n = 3 donors) as a control (Fig. 3G, Additional file 3: Fig. S6B, D and Additional file 4: Fig. S7C–D). First it was noticeable that human DM samples had some areas with mesenchymal-like cell growth underneath the CEnC-like cells in both conditions (Additional file 4: Fig. S7). Interestingly, CD166 stainings indicated that the cells injected with bioink had stronger CD166 expression as compared to the control cells seeded without bioink (Fig. 3F–G). Nevertheless, when investigating this further, the CD166 expression levels between hPSC-CEnCs injected with bioink and seeded without bioink did not show statistical difference in RT-qPCR analysis when samples were cultured on cell culture plastics (Additional file 5: Fig. S8A).

### Bioink promotes higher hPSC-CEnC density and smaller cell size

**Fig. 4:**
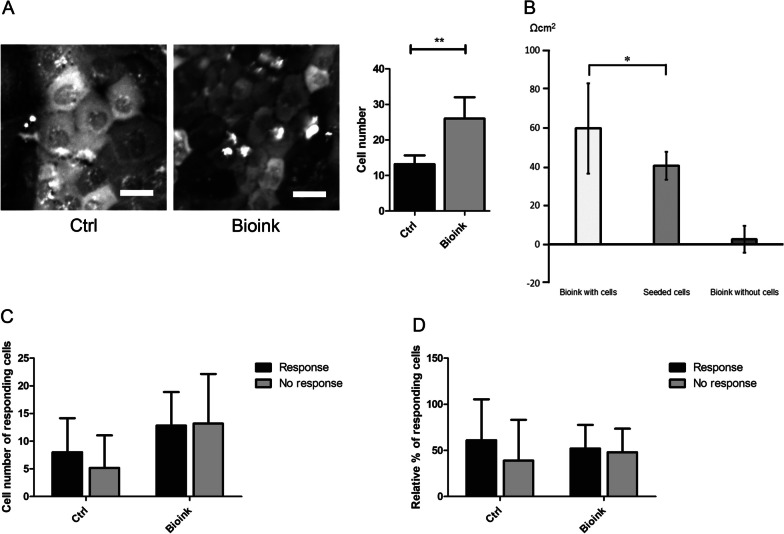
The effects of bioink on hPSC-CEnC cell size, barrier and Ca^2+^-signaling properties after 6 days of injection/seeding. Experiments conducted with the hESC line. **A** Cell number analysis between bioink injected cells and seeded cells with representative ROI-images (n = 6 per sample type) from which the cell number calculations were measured. **B** TEER value measurements of the hPSC-CEnCs injected with bioink, seeded without bioink (n = 4 per sample type) and bioink without cells (n = 3). TEER value of empty insert (n = 4) was subtracted from the values. All samples were treated with identical medium changes. **C** Cell number of responding hPSC-CEnCs injected in bioink and seeded without bioink (ctrl). **D** Relative percent of responding hPSC-CEnCs injected in bioink and seeded without bioink (ctrl). All data represents mean ± SD. A) **p = 0.002, B) *p < 0.05, no significant difference between other samples, **C–D** no significant difference was detected. ROI-image size 200 × 200 pixels taken with 25 × objective. Scale bars 20 µm (**A**)

The ZO-1 staining (Fig. 3F–G) indicated that the cells injected with bioink were smaller as compared to the control cells seeded without bioink. It was further observed in later analyses that the cell number in the bioink samples was also higher indicating higher cell density and smaller cells as compared to the seeded controls (Fig. 4A, Additional file 5: Fig. 8B). The cell size analysis with NucleoCounter® NC-200™ (with both used cell line) further confirmed smaller average cell diameter for hPSC-CEnCs injected with bioink as compared to seeded culture (Additional file 5: Fig. S8B).

### Developed bioink demonstrates enhanced functional characteristics of hPSC-CEnCs

The barrier integrity and functionality of the hPSC-CEnCs with bioink was tested with TEER measurements. The TEER value of hPSC-CEnCs injected with bioink (59.8 ± 23.2 Ωcm^2^) was significantly (p < 0,05) higher compared to seeded controls (40.5 ± 7.1 Ωcm^2^). The value of inserts with bioink without cells (2.7 ± 7.0 Ωcm^2^) were close to the value of empty inserts (0 ± 2.9 Ωcm^2^) (Fig. 4B). The Ca^2+^-imaging was used to confirm the equal ATP-induced Ca2 + responses between bioink injected and seeded hPSC-CEnCs without statistically significant difference (Fig. 4C–D).

### Developed bioink demonstrates high cytocompatibility with hPSC-CEnCs in bioprinting

**Fig. 5:**
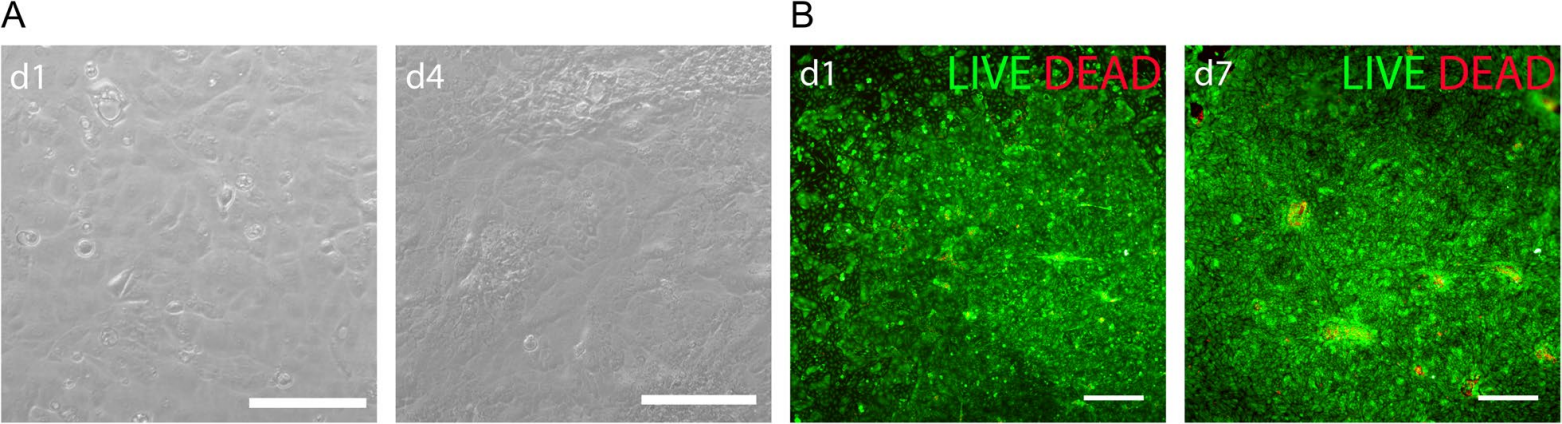
The bioink shows high cytocompatibility with bioprinted hPSC-CEnCs. Experiments conducted with the hESC line. **A** Representative phase contrast images of bioprinted hPSC-CEnCs on a fibrin membrane after 1 and 4 days of bioprintig (n = 3). Scale bars 200 µm. **B** Representative images of the viability of hPSC-CEnCs after 1 and 7 days of bioprinting analyzed with live-dead-staining (n = 3). Scale bars 500 µm

The cytocompatibility of the bioink was further tested with bioprinting of hPSC-CEnCs on fibrin membrane. Phase contrast microscopy was used to monitor the morphology of the printed hPSC-CEnCs at 1 and 4 days after printing confirming their normal polygonal morphology (Fig. 5A). There was no difference if the fibrin membrane was coated or noncoated with LN521™ (data not shown). To analyze the cytocompatibility of the bioink with hPSC-CEnCs, LIVE/DEAD® viability/cytotoxicity kit with bioprinted hPSC-CEnCs at day 1 and day 7 time points was used demonstrating high viability of the hPSC-CEnCs after bioprinting (n = 3) (Fig. 5B, Additional file 6: Fig. 9A–B). Calculated average percentage of dead cells at day 1 was 7.5% (ROI = 3). The number of dead cells did not increase substantially upon culture of 7 days.

### Bioprinting of cornea endothelium mimicking tissue equivalent using hPSC-CEnCs

**Fig. 6:**
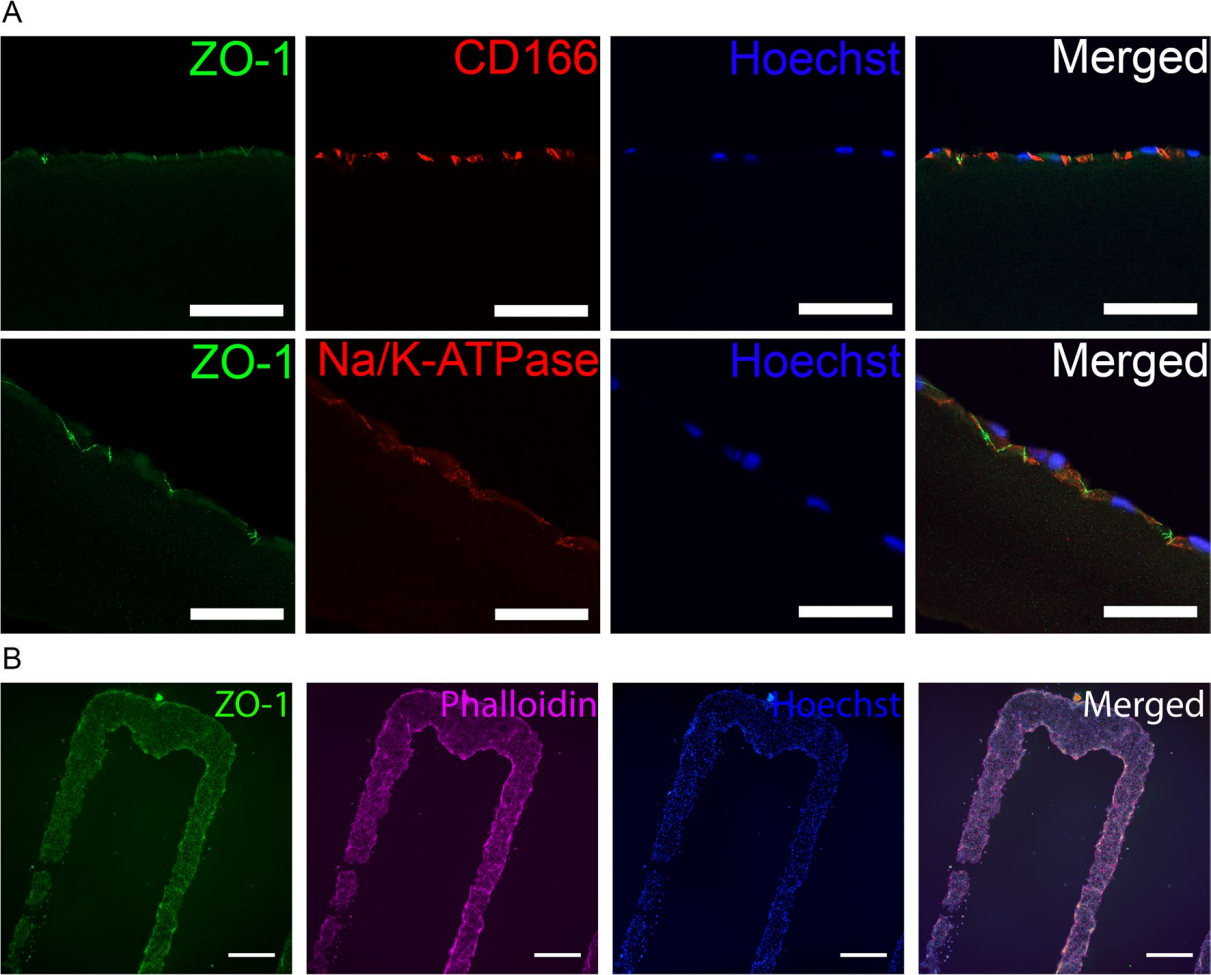
Immunofluorescence stainings of monolayered bioprinted hPSC-CEnCs on fibrin membrane and the patternability of the bioink. Experiment conducted with the hESC line. **A** Representative confocal microscope images of cryosections stained with ZO-1, CD166, Na^+^/K^+^-ATPase and Hoechst 7 days after printing on fibrin membrane (n = 1). **B** Representative images of hPSC-CEnCs printed in a line pattern with a 1.3 mm line distance on cell culture plastic after 1 day (n = 2). ZO-1 (green), Phalloidin (purple) and Hoechst (nuclei, blue). Scale bars 50 µm (**A**), 500 µm (**B**)

The developed 3D bioprinting parameters were successfully used to fabricate cornea endothelium mimicking structure on fibrin membrane. Immunofluorescence images from cryosections showed a monolayer of hPSC-CEnCs with localized expression ZO-1, CD166 and Na^+^/K^+^-ATPase (Fig. 6A). The ZO-1 and CD166 expression resembled the rat and porcine ex vivo sections (Additional file 1: Fig. S4). Furthermore, the hPSC-CEnCs were successfully printed into lines with precise placement with line distance of 1.3 mm according to the initial CAD design (Fig. 6B), indicating that the bioink allows precise spatial placement of cells into complex constructs.

**Fig. 7:**
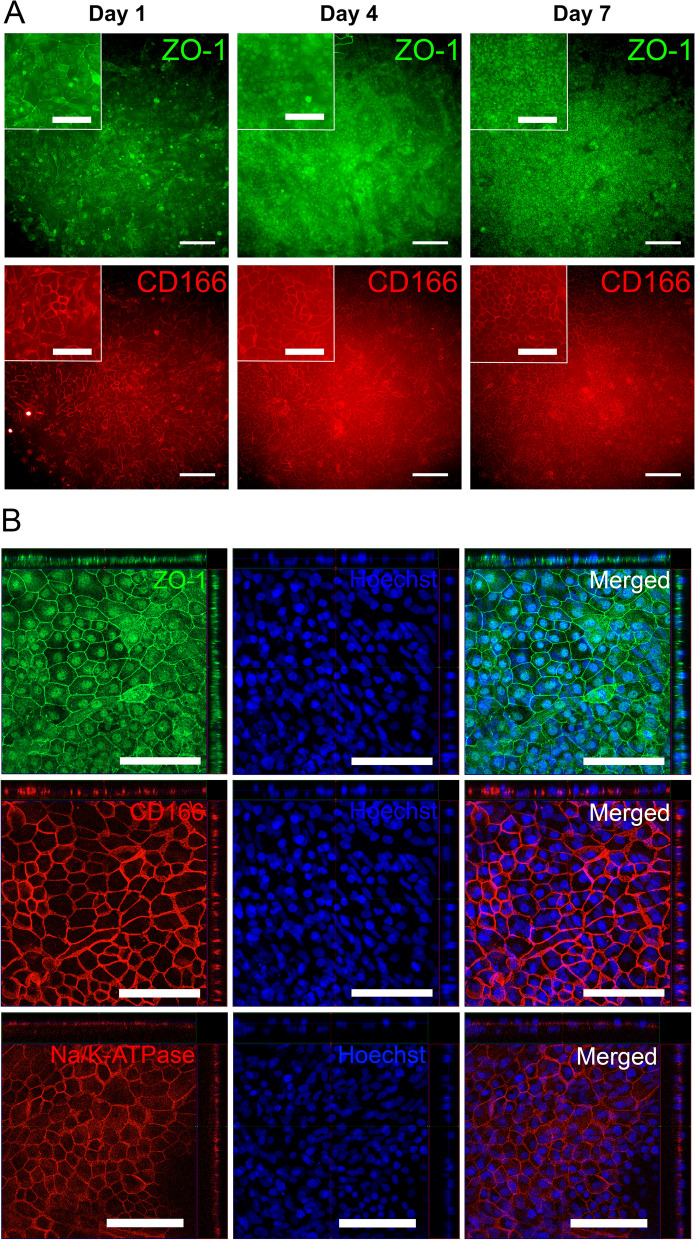
Immunofluorescence stainings of bioprinted hPSC-CEnCs on fibrin membrane. Cells were bioprinted in cylindrical structures of two printed layers with 0.30 mm line distance. Experiment conducted with the hESC line. **A** Representative fluorescence microscope images of ZO-1 (green) and CD166 (red) expression from day 1, 4 and 7 after printing (n = 2 for every timepoint). Relatively thick fibrin membrane produces background noise to the images. **B** Representative confocal microscope ortho images of ZO-1 (green), CD166 (red), Na^+^/K^+^-ATPase (red) and Hoechst (blue) expression at day 7 after bioprinting (n = 2). Scale bars **A** 200 µm (in inserted magnified images 100 µm), **B** 100 µm

For bioprinting uniform cornea endothelium, cylindrical structures with smaller line distance of 0.30 mm were used. Representative images of the printed structures are illustrated in Additional file 7: Fig. S10. After the bioprinting, the morphology of hPSC-CEnCs improved from day 1 to day 7 (Fig. 7A, Additional file 8: Fig. S11) and immunofluorescence microscopy showed that the cells became more hexagonal, and they demonstrated localization of ZO-1 in the tight junction areas. Typical CEnC marker CD166 was expressed throughout the 7 days follow-up after bioprinting. When important CEnC markers were further analyzed with confocal microscopy at day 7, the expression of crucial pump protein Na^+^/K^+^-ATPase was evident in the basolateral side of the cells demonstrating polarization of the endothelium (Fig. 7B). Interestingly, the hPSC-CEnCs printed on fibrin membrane compared to seeded hPSC-CEnCs on fibrin membrane showed no prominent difference in morphology or in ZO-1 and CD166 protein expression (Additional file 9: Fig. S12A–B). Even though the hPSC-CEnCs formed solid layer of hexagonal cells with round (from above) nucleus, some unidentified cells with elliptical nucleus were proliferating underneath the hPSC-CEnCs in certain areas that could be verified with Ki67 staining (Additional file 9: Fig. S12C–D).

## Discussion

Corneal transplantation is the state-of the-art therapy for corneal endothelial diseases but the increase in transplantations causes a global donor tissue shortage [1]. Human CEnCs in native cornea are organized in a tight mosaic of hexagonal cells and serve a critical function in maintaining corneal hydration and clear vision. The main barriers for the in vitro culture of CEnCs are the difficulties of forcing quiescent cells to proliferate while avoiding endothelial to mesenchymal transition, which would lead to a cellular transdifferentiation towards a myofibroblastic phenotype causing a cellular loss of function [8]. Unfortunately, primary CEnCs even from young donors can only be passaged a few times before they present genetic and functional alterations, which limits the number of cells that can be generated from a single donor cornea [34–36].

To tackle the current tissue shortage and the limitations for the in vitro expansion of primary CEnCs, the de novo generation of CEnCs from hPSCs or other cell sources is needed in addition to the advanced and standardized cell and tissue production methods for clinical applications. In this concept, both the novel source of CEnCs from hPSCs [16–18, 21–25] and 3D bioprinting of partial or full thickness corneal equivalent including all three cell layers (endothelium, stroma and epithelium) would be the holy grail in corneal tissue engineering and regenerative medicine [37]. Various studies have used 3D bioprinting for corneal tissue, concentrating mainly on stroma and epithelium, but corneal endothelium has received less attention [9–11]. In principle, the low thickness of corneal endothelium and the lack of vascularization makes it an ideal target for bioprinting but the low proliferation rate of CEnCs brings additional challenges including issues with viability of these dedicated cells after the bioprinting process. To our knowledge, only one study with 3D bioprinted corneal endothelium has been carried out by Kim and co-authors [15] where they introduced an extrusion-based 3D bioprinting method to create corneal endothelium structures. Instead of using hPSC-CEnCs, they used genetically modified human primary CEnCs (overexpression of ribonuclease 5) to improve proliferation and functionality of the primary cells. In their study, these cells were embedded in a gelatin-RGD bioink and 3D bioprinted on the lyophilized and decellularized bovine amniotic membrane [15]. There are some identified integration concerns related to the gelatin based materials as a scaffolds for CEnCs as previous work has reported that four weeks after implantation, 60% of the transplanted scaffolds detached from the recipients’ corneal stroma [38]. Thus, instead of using gelatin based bioink or animal-based printing substrate, we used clinically suitable and biodegradable HA as a base of the bioink and synthetically prepared fibrin surface as bioprinting substrate for the hPSC-CEnCs. Importantly, novel solutions for clinically compatible CEnCs supportive bioink was developed and tested together with hPSC-CEnCs.

Bioinks with adequate structural strength are thought to be ideal for printing of corneal stromal alternatives to achieve the required biomechanical functions of the cornea. In contrast, bioinks suitable for bioprinting corneal endothelium depend less on the mechanical properties, but more on the biocompatibility as a cell-carrier for the manufacturing process and cell patterning with high viability [11]. Here together with lower viscosity characteristics, human Col IV and recombinant LN521™ were used as additional rheological modifiers in the bioink and to improve the cytocompatibility and functionality of the bioink to support the delicate hPSC-CEnCs. The selection of extracellular matrix proteins was guided by the knowledge that DM is enriched with type IV collagen and laminin α5 [39] and based on our previous results that LN521™ supports the differentiation and culture of hPSC-CEnCs [16]. First, we analyzed the shear-thinning properties with viscosity measurements for the bioink with and without cells and demonstrated that only slight but not significant increase was seen in the viscosity of the bioink with cells. The effect of cells on the bioink’s viscosity has not been widely studied, even though viscosity and shear-thinning properties are crucial features for the printability in extrusion-based bioprinting. Some studies have shown an increase in viscosity when adding cells to the bioink [40–43], and others have observed a decrease [44–46]. Therefore, this link between the cell density and the viscosity is not yet fully understood, and further research is required. Even though a slight difference in viscosities between bioinks with and without cells was observed in our study, it did not affect the printability of the cell containing bioink. Similar results have been observed by Gillispie and co-workers and they also suggested that rheological measurements in general are more sensitive to detect changes in bioink properties as compared to the printability assessment methods [40]. In our study, despite its relatively low viscosity, the bioink showed good printability with 32G nozzles and demonstrated formation of continuous filaments and grid structures in the optimized printing process. Importantly, bioink demonstrated good shape fidelity in culture 7 days after printing. Slight decrease in filament thickness as well as pore factor was detected during the 7-day culture period time. In addition, the swelling test demonstrated high decrease of material with only 26% of the weight of the material left at day 10. This decrease of material is mostly due to the removal of free HA, collagen and laminin added to the bioink formulation to improve the rheological properties of the bioink and their leakage from the material during culture [14]. In the bioink composition used in this study, the relative percentage of weight of the non-crosslinking rheological components compared to the crosslinking components was high resulting in high loss of material in the swelling and degradation study. Despite the loss of material due to leaking out of the crosslinking components, the printed structures maintained their grid like structures well in vitro demonstrating good shape fidelity.

The biocompatibility of the bioink with hPSC-CEnCs was confirmed with functional assessment of the endothelial barrier integrity with TEER measurements, followed by ATP-induced Ca.^2+^ responses analyses between bioink injected and seeded hPSC-CEnCs. Both used methods confirmed functionality of the cells with the bioink, TEER values being even higher with bioink injected cells as compared to the seeded cells. Other researchers have suggested in recent studies that ATP-induced calcium signals may have a significant impact on regulating apoptosis in endothelial cells, even in non-injured states [47]. Consequently, this suggests that such signals could serve as a valuable tool for evaluating the functionality of endothelial cells and thus could be used as a valuable tool for the functional assessment of endothelial cells. In previous studies, we have employed same methods to assess the quality and functionality of retinal pigment epithelial cells as well. [34–36]

Next, we studied the biocompatibility and integration of the bioink with hPSC-CEnCs by using our previously established ex vivo porcine cornea model [27] to avoid unnecessary use of experimental animals. In addition, ex vivo analyses with rat corneas enabled also flat mount analyses of the integrated cells. With verification of using human specific antibody STEM121, the successful integration of hPSC-CEnCs to host DM was confirmed with typical CEnC characteristics demonstrated with ZO-1 and CD166 expression and localization. Importantly, the high tissue integration of the bioink with cells was evident without additional functionalization of the bioink such as using RGD-peptide [15]. Bioinks that can achieve adequate adhesion between bioprinted constructs and host tissue surfaces, in particular surfaces that are oftentimes wet, have hardly been investigated [48]. In our study, the advantage of the tissue adhesive capability of dopamine, a catecholamine derived from marine mussels that functions as a glue in wet conditions, was introduced to the bioink by covalently grafting dopamine on HA [14, 27]. The high tissue integration of the bioink to host DM indicates that the bioink might have adhesive properties also in this low viscous composition. However, additional studies are needed to confirm these findings also in vivo. Interestingly, the biocompatibility and integration of the bioink with hPSC-CEnCs was confirmed with human DM and the ZO-1 and CD166 stainings which indicated that the cells injected with bioink were smaller and had increased CD166 expression with cell membrane localization as compared to the control hPSC-CEnCs seeded without bioink. Although, the CD166 expression analyzed with RT-qPCR did not show significant difference between bioink and seeded samples this is most likely is due to the heterogeneity of the cells as well as substrate differences as the immunostainings were conducted on the top of the DM as compared to the RT-qPCR quantification conducted from the samples on cell culture plastics. However, further analyses confirmed the results that the bioink supported the higher cell density integration and smaller cell size in all culture substrates as compared to the seeded cells. One possible reason for these observations could be the higher contact inhibition with bioink injected cells which is also supported by the increased TEER. But further research is needed to draw any justified conclusions. Nevertheless, these results suggest that the bioink provides adequate support for the CEnC phenotype with successful integration to the host tissue, although some unwanted mesenchymal-like cell growth underneath the CEnC-like cells both with and without bioink was observed. This observation suggests bioink independent issues with the purity of the used hPSC-CEnCs that we have observed here as well as in our previous studies with in vitro cultured hPSC-CEnCs [16]. The positive expression of keratocan and PAX6 already before bioprinting among hPSC-CEnCs indicates contaminating cells representing possible corneal stromal cells and other eye-related cells [49]. Further development of the differentiation method efficacy or identification of additional specific cell surface markers for CEnC purifications is essential future directions needed in the field.

Last important part of our study was to demonstrate the functionality of the developed bioink in bioprinting of hPSC-CEnCs. Due to the small printing nozzle, low viscosity bioink for bioprinting with low printing pressure was used to reduce shear stress and prevent the cell death [50]. The high viability of hPSC-CEnCs after printing demonstrated a good cytocompatibility of the bioink with cells during the printing process. Importantly, the developed 3D bioprinting parameters were successfully used to fabricate cornea endothelium mimicking structure on fibrin membrane. With immunofluorescence analyzes a monolayer of hPSC-CEnCs with correctly localized expression ZO-1 and CD166 was confirmed. Importantly, clear signs of the further endothelium maturation after bioprinting were evident with improved CEnCs-like hexagonal cell morphology at day 7 as compared to day 1. Immunofluorescence analyzes further confirmed the presence of tight junctions with ZO-1 expression and the expression of Na^+^/K^+^-ATPase was also evident in the basolateral side of the cells 7 days after bioprinting. These results provide the first proof of concept of the successful bioprinting of human endothelium mimicking structures with low viscose and hPSC-CEnC containing bioinks.

As a final note, despite the current advances in primary culture of CEnCs and clinical trials ongoing with injection of these cells [51], many questions remain unresolved including donor-dependent variability, need of the young donors and few studies showing the biodistribution of injected CEnCs and the effect they may have both within the recipient’s eye and systemically with possible safety concerns [8]. Although cell injections are used in the delivery of primary CEnCs to the anterior chamber of the eye as a simple and minimally invasive manner, the injection is not yet an efficient method although gravity (patient lie face down after operation), for example, has been shown to increase CEnC adherence to the posterior part of the cornea. Thus, improving cell adherence and survival during this procedure would reduce the number of cells needed to treat one diseased cornea, allowing more patients to benefit from this technique. As our results clearly indicated successful integration of hPSC-CEnCs to host DM (rat, porcine and human) ex vivo with the developed bioink, it is tempting to consider that the bioink (with trace of tissue-adhesive properties due to dopamine [14]) could provide an appealing strategy for CEnC injections and delivery of cells to the correct place in a controlled manner with enhanced tissue integration. Certainly, additional research including in vivo studies is required to investigate the applicability of the 3D bioprinting method for both partial and full-thickness corneas, as well as for applications where bioink would be used as a carrier for injecting CEnC.

## Conclusion

In conclusion, by using our developed simple and directed differentiation protocol for hPSC-CEnCs and cornea endothelium specific bioink design, we successfully demonstrated high tissue integration of the cell containing bioink and proof-of-concept for the bioprinted natural corneal endothelium resembling structure. The bioprinted hPSC-CEnCs demonstrated high cell viability and a polygonal, tightly packed cell morphology with expression of proteins important for functional CEnCs. This study is the missing part of engineering of human stem cell-based 3D bioprinted full-thickness cornea as epithelium and stroma has been already successfully bioprinted in our previous research [10]. The partial and full-thickness 3D bioprinted cornea with functional endothelium, could be easily tailored based on clinical needs of the patient. With automated and highly standardized advanced manufacturing technologies and scalable hPSCs, this could be the future for treating patients suffering from corneal blindness.

### Supplementary Information

**Additional file 1 Fig. S4 **Immunofluorescence images of hPSC-CEnCs on ex vivo rat (n=1 rat with total of 2 corneas) and porcine cornea (n=2 pigs with total of 2 corneas). **A–B** Representative images of hPSC-CEnCs in bioink injected on rat cornea at day 5. **C–D** Representative images of hPSC-CEnCs seeded on rat cornea without bioink at day 5. **E** Representative images of IgG controls of hPSC-CEnCs seeded on rat cornea without bioink at day 5. **F–G** Representative images of hPSC-CEnCs in bioink injected on porcine cornea at day 10. Below the dashed line is stroma and above DM with hPSC-CEnCs on it. One headed arrow indicates the region of the magnified image, two headed arrows indicate the thickness of porcine DM. In **A**, **C**, **F** ZO-1 (green), STEM121 (red) and Hoechst (blue); **B**, **D** Na+/K+-ATPase (green) CD166 (red) Hoechst (blue); **E** IgG control for Alexa Fluor 488 (green), IgG control for Alexa Fluor 568 (red) and Hoechst (blue); G) ZO-1 (green) CD166 (red) and Hoechst (blue). Scale bars 100μm (**A–E**), 50 μm but in magnified images 6.25 μm (**F–G**).

**Additional file 2 Fig. S5** Representative images of tissue integration demonstrated with H&E staining. hPSC-CEnCs injected with bioink on the Descemet’s membranes of rat at day 5 (n=1 cornea) **A** and porcine ex vivo corneas at day 10 (n=1 cornea) **B**. Scale bar 50 μm.

**Additional file 3 Fig. S6 **Representative immunofluorescence images of hPSC-CEnCs on human DM (n=6 donors) injected with bioink (n=3) or seeded without bioink (n=3) at day 6 showing CEnC-like characteristics with ZO-1, CD166 and Na+/K+-ATPase stainings but also proliferation of unwanted cells with Ki67 stainings. Experiments conducted with the used hESC line. **A–B** Stained with ZO-1 (green), CD166 (red) and Hoechst (blue); **C–D** Stained with Ki67 (green), Na+/K+-ATPase (red) and Hoechst (blue); **E** IgG control for Alexa Fluor 488 (green), IgG control for Alexa Fluor 568 (red) and Hoechst (blue). Scale bars 200 μm.

**Additional file 4 Fig. S7 **Representative immunofluorescence stainings showing mesenchymal-like cell growth beneath the CEnC-like cell layer. Experiments conducted with the used hESC line. **A**, **B** Confocal microscope stack images of hPSC-CEnCs containing bioink injected on human DM (n=3 donors) and **C**, **D** hPSC-CEnCs without bioink cultured for 6 days on human DM (n=3 donors). **A** and **C** are imaged from the top cell layer with hPSC-CEnCs and **B** and **D** are imaged from the middle cell layer with mesenchymal-like cells visible. Scale bars 20 μm. In **A**–**D** ZO-1 (green), CD166 (red) Hoechst (cyan); Scale bars 20 μm (**A–D**).

**Additional file 5 Fig. S8 **Additional quantitative characterizations of the hPSC-CEnCs using both of the used hPSC lines. **A** RT-qPCR analysis of the CD166 (ATCAM) expression between hPSC-CEnCs injected with bioink and seeded without bioink with Regea08/017 hESC line and WT001.TAU.bB2 hiPSC line (n=3 technical replicates from each). **B** Cell number quantification between bioink injected and seeded hPSC-CEnCs. Analyses conducted manually from ROIs (500 x 500 pixels) including 30 ROIs for bioink injected and 30 ROIs seeded cells (Regea08/017 line) and 13 ROIs for bioink injected and 13 ROIs for seeded cells (WT001.TAU.bB2 line). Cell diameter measured with NucleoCounter® NC-200™ (Regea08/017 bioink n=26 cells and seeded n=7 cells; WT001.TAU.bB2 bioink n=190 cells and seeded n=89 cells). All data represents mean ±SD except no SD for cell diameter measurement. In **A** no significant difference (p=0.936 for Regea08/017 and p=0.227 for WT001.TAU.bB2) was detected, in **B** ***p<0.001. ROI-image size 500x500 pixels taken with 10x objective.

**Additional file 6 Fig. S9 **Viability of bioprinted hPSC-CEnCs differentiated from the hESC line and data verifications with additional hiPSC line. **A–B** Representative images of the viability of bioprinted hPSC-CEnCs differentiated from two individual hESC line batches from day 1 (n=2 per batch) and day 7 (n=2 per batch). **C** Representative image of the viability of bioprinted hPSC-CEnCs differentiated from hiPSC line from day 1 (n=2) and day 7 (n=2) **D** immunofluorescence images of bioprinted hPSC-CEnCs differentiated from hiPSC cell line (n=1). ZO-1 (green), CD166 (red) and DAPI (blue). Scale bars 400μm (**A–C**) and 200 μm (**D**).

**Additional file 7 Fig. S10 **Representative images of the printed structures for bioprinting hPSC-CEnCs. **A** For printing cells, line distance of 0.30 mm was used, resulting in filament fusion and uniform bioink layer in the first layer. **B** Bioprinted structure after 2nd printed layer imaged with a high-definition CCD-camera attached to the dispenser head mount immediately after printing. Scale bars 10 mm. **C** A representative two-layered bioprinted structure used for hPSC-CEnCs printing on a cell culture dish

**Additional file 8 Fig. S11 **Additional representative immunofluorescence stainings of bioprinted hPSC-CEnCs from day 1 (n=1) and day 7 (n=2) stained with ZO-1 (green), CD166 (red) and Hoechst (blue). Experiments conducted with the used hESC line. Scale bar 200 μm.

**Additional file 9 Fig. S12 **Representative immunofluorescence images of bioprinted **A** and seeded **B** hPSC-CEnCs on fibrin membrane at day 7 (n=2). Experiments conducted with the used hESC line. Cell cultures are showing similar characteristics with ZO-1 and CD166 markers. **C** Immunofluorescence stainings of proliferation marker Ki67 shows growth of unwanted cells in the hPSC-CEnC culture in the bioprinted 7 days after bioprinting (n=1) and **D** also in the seeded hPSC-CEnC control on fibrin membrane (n=1). In A-B) ZO-1 (green), CD166 (red) and Hoechst (blue). Scale bars 100 μm, magnified image 50μm. In **C–D** Ki67 (green) Hoechst (blue). Scale bars 400 μm.

**Additional file 10 Fig. S1 **All data points of **A** Filament thickness measurements (number of data points per time point 54) and **B** Pore factor (number of data points per time point 18).

**Additional file 11 Fig. S2 **A representative original image used for the printability and shape fidelity analysis of the bioink.

**Additional file 12 Fig. S3 **Characterization of differentiated hPSC-CEnCs used for bioprinting. **A** Representative immunofluorescent images of thawed hPSC-CEnCs stained with CD166 (red) and DAPI (blue) differentiated from Regea08/017 cell line and **B** WT001.TAU.bB2 cell line. **C** FC analysis of hPSC-CEnCs (WT001.TAU.bB2 cell line) stained with CD166 from day 10 of differentiation. **D** Representative phase contrast image of hPSC-CEnC (Regea08/017 cell line) culture before bioprinting with cell islands of unwanted cells. **E** Representative immunofluorescent image of hPSC-CEnCs stained with keratocan (purple) and DAPI (blue) differentiated from Regea08/017 line showing possible keratocyte-like cells in the cell culture. **F** Representative image of PAX6 (green) positive cells amongst the unwanted cell population. Merged with DAPI (blue). Scale bars 400 μm (**A–B**, **E–F**) and 200 μm (**D**).

## Data Availability

All the data used to support the findings of this study are included within the article and its additional files.

## Abbreviations

ALD: Aldehyde
BSA: Bovine serum albumin
CDH: Carbodihydrazide
CEpC: Corneal epithelial cells
CHIR: CHIR99021
Col IV: Collagen IV
DA: Dopamine
DAPI: 4’,6-Diamidine-2’-phenylindole dihydrochloride
DM: Descemet membrane
DSEK: Descemet stripping endothelial keratoplasty
DMEK: Descemet membrane endothelial keratoplasty
DMSO: Dimethyl sulfoxide
DPBS: Dulbecco’s Phosphate Buffered Saline
DTI: Defined trypsin inhibitor
EDTA: Ethylenediamine-tetraa cetic acid
FC: Flow cytometry
HA: Hyaluronic acid
hCEnC: Human corneal endothelial cells
hESC: Human embryonic stem cell
hiPSC: Human induced pluripotent stem cell
KO-DMEM: KnockOut Dulbecco’s Modified Eagle Medium
KO-SR: Knock-out serum replacement
LN521: Laminin 521
Na^+^/K^+^ ATPase: Sodium–potassium pump
PFA: Paraformaldehyde
Pr: Pore factor
RA: Retinoic acid
ROCKi: ROCK inhibitor Y27632
ROI: Region of interest
SB: SB431542
Stl: Stereolithography
TEER: Transendothelial electrical resistance
ZO-1: Zona Occludens-1
