# Supplementary figures and images for: Bioprinting of human pluripotent stem cell derived corneal endothelial cells with hydrazone crosslinked hyaluronic acid bioink
Source: Stem Cell Res Ther. 2024 Mar 14;15:81. doi: 10.1186/s13287-024-03672-w (PMC10941625; doi:10.1186/s13287-024-03672-w)

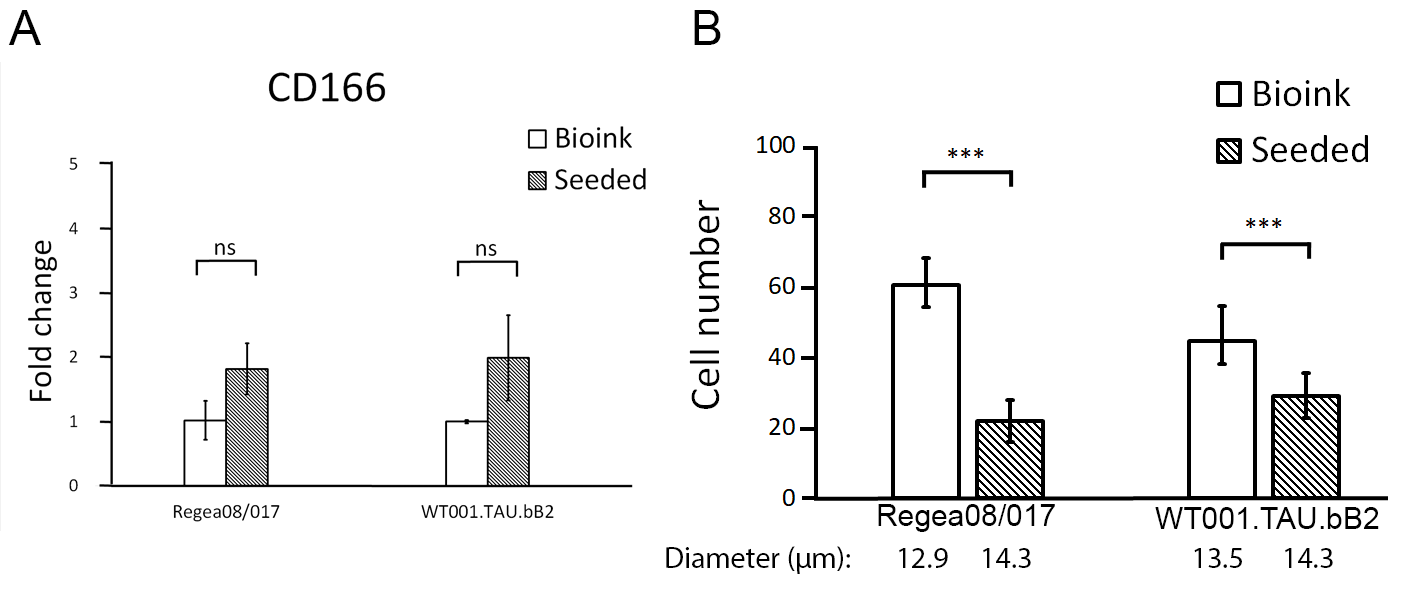

Supplement: Supplementary file 2 — Additional file 2 Fig. S5 Representative images of tissue integration demonstrated with H&E staining. hPSC-CEnCs injected with bioink on the Descemet’s membranes of rat at day 5 (n=1 cornea) A and porcine ex vivo corneas at day 10 (n=1 cornea) B. Scale bar 50 μm. [file 13287_2024_3672_MOESM2_ESM.tif]

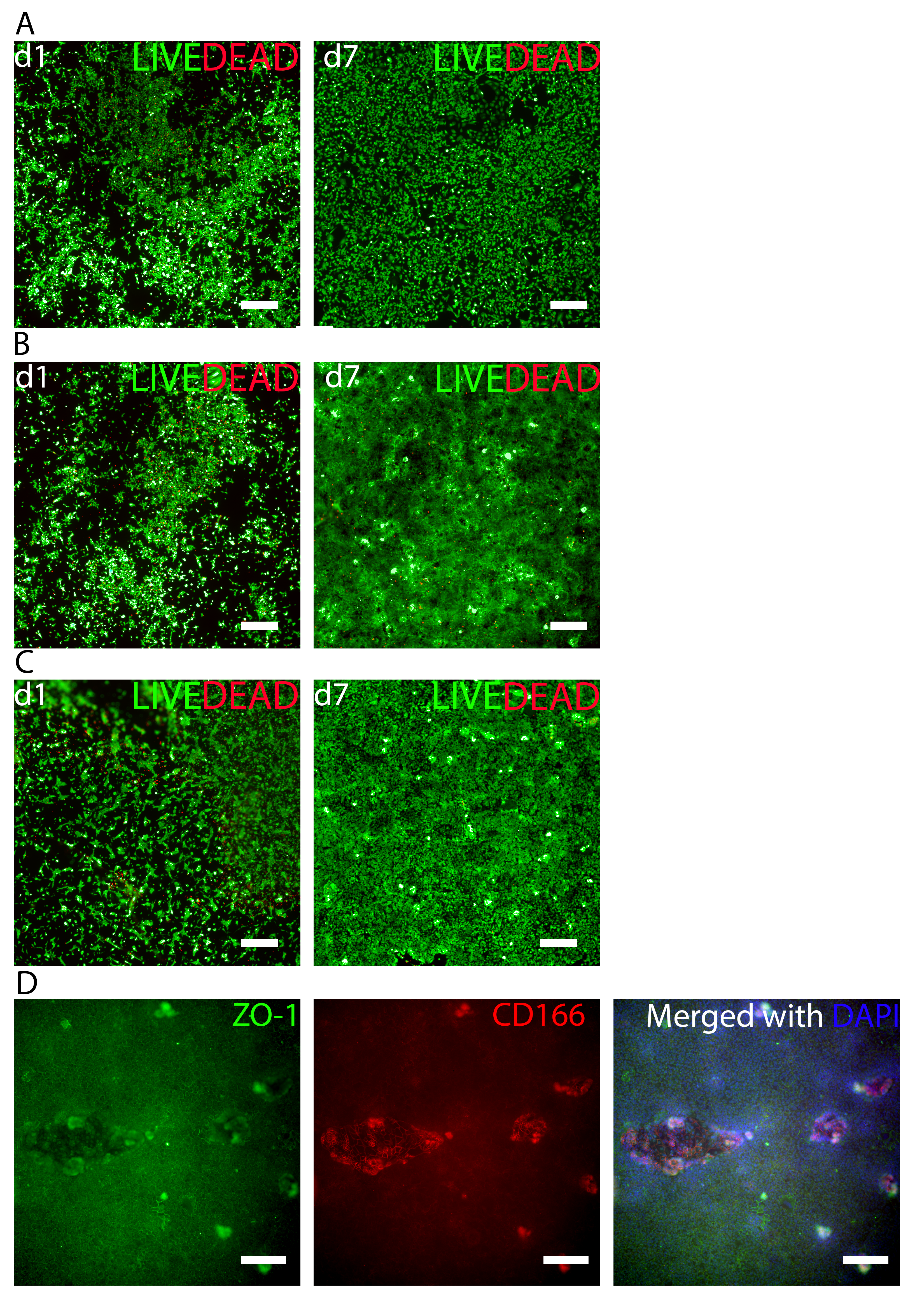

Supplement: Supplementary file 3 — Additional file 3 Fig. S6 Representative immunofluorescence images of hPSC-CEnCs on human DM (n=6 donors) injected with bioink (n=3) or seeded without bioink (n=3) at day 6 showing CEnC-like characteristics with ZO-1, CD166 and Na+/K+-ATPase stainings but also proliferation of unwanted cells with Ki67 stainings. Experiments conducted with the used hESC line. A–B Stained with ZO-1 (green), CD166 (red) and Hoechst (blue); C–D Stained with Ki67 (green), Na+/K+-ATPase (red) and Hoechst (blue); E IgG control for Alexa Fluor 488 (green), IgG control for Alexa Fluor 568 (red) and Hoechst (blue). Scale bars 200 μm. [file 13287_2024_3672_MOESM3_ESM.tif]

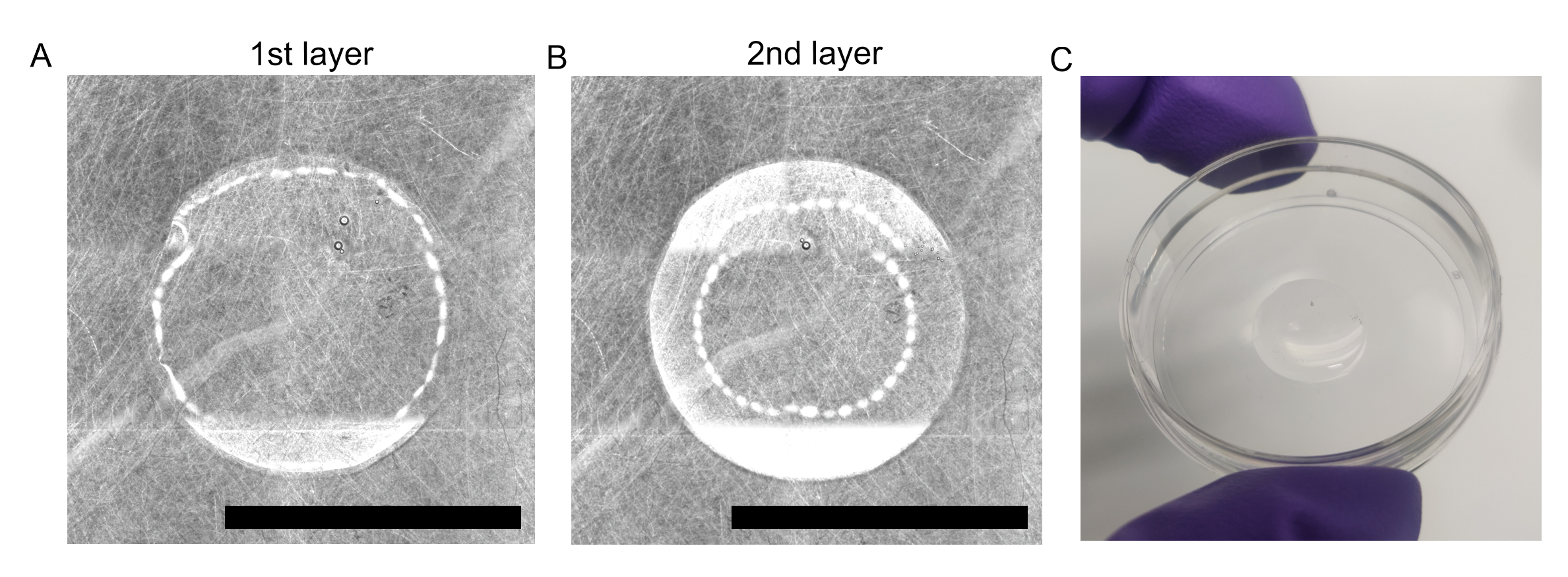

Supplement: Supplementary file 4 — Additional file 4 Fig. S7 Representative immunofluorescence stainings showing mesenchymal-like cell growth beneath the CEnC-like cell layer. Experiments conducted with the used hESC line. A, B Confocal microscope stack images of hPSC-CEnCs containing bioink injected on human DM (n=3 donors) and C, D hPSC-CEnCs without bioink cultured for 6 days on human DM (n=3 donors). A and C are imaged from the top cell layer with hPSC-CEnCs and B and D are imaged from the middle cell layer with mesenchymal-like cells visible. Scale bars 20 μm. In A–D ZO-1 (green), CD166 (red) Hoechst (cyan); Scale bars 20 μm (A–D). [file 13287_2024_3672_MOESM4_ESM.tif]

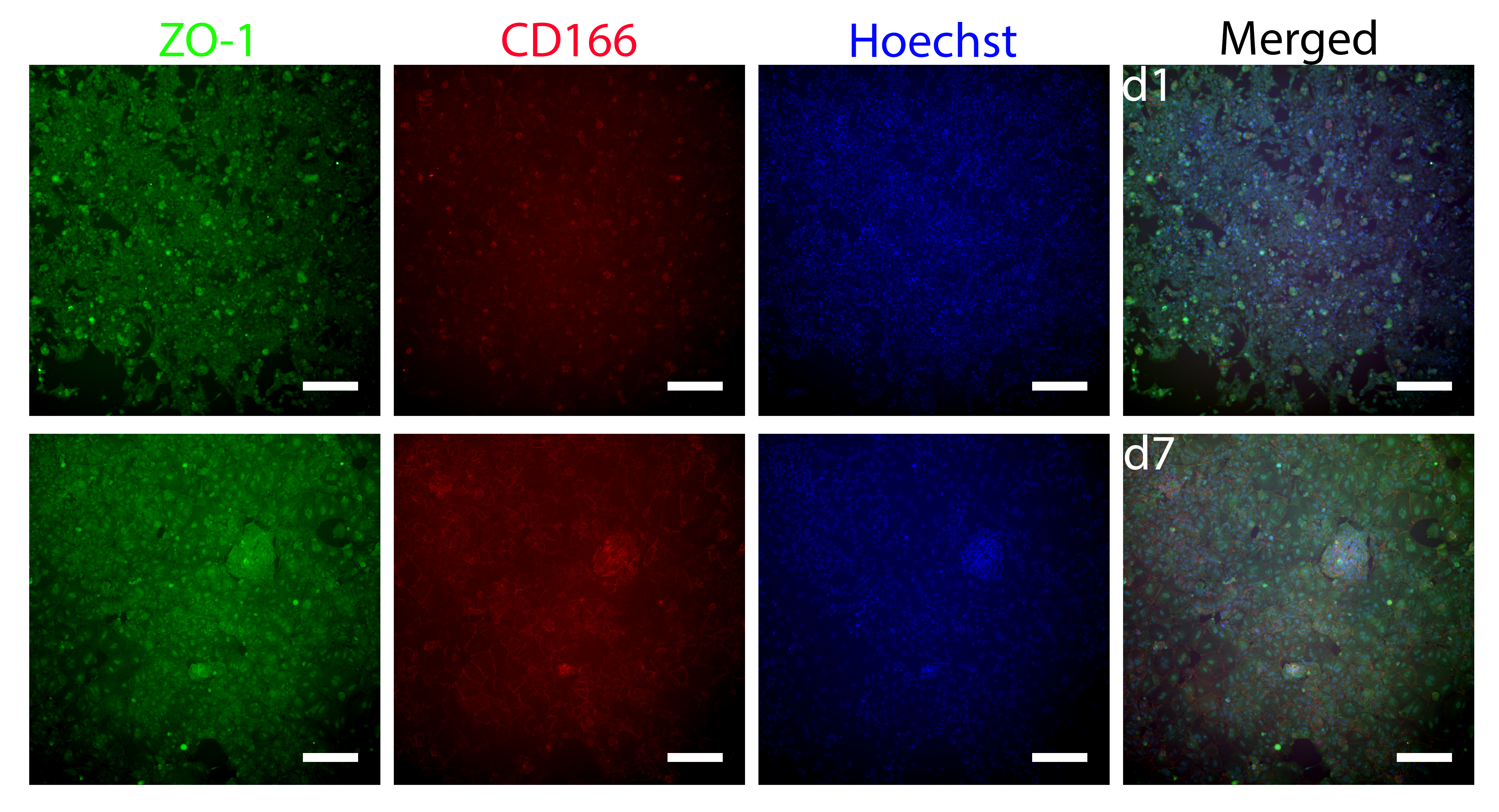

Supplement: Supplementary file 5 — Additional file 5 Fig. S8 Additional quantitative characterizations of the hPSC-CEnCs using both of the used hPSC lines. A RT-qPCR analysis of the CD166 (ATCAM) expression between hPSC-CEnCs injected with bioink and seeded without bioink with Regea08/017 hESC line and WT001.TAU.bB2 hiPSC line (n=3 technical replicates from each). B Cell number quantification between bioink injected and seeded hPSC-CEnCs. Analyses conducted manually from ROIs (500 x 500 pixels) including 30 ROIs for bioink injected and 30 ROIs seeded cells (Regea08/017 line) and 13 ROIs for bioink injected and 13 ROIs for seeded cells (WT001.TAU.bB2 line). Cell diameter measured with NucleoCounter® NC-200™ (Regea08/017 bioink n=26 cells and seeded n=7 cells; WT001.TAU.bB2 bioink n=190 cells and seeded n=89 cells). All data represents mean ±SD except no SD for cell diameter measurement. In A no significant difference (p=0.936 for Regea08/017 and p=0.227 for WT001.TAU.bB2) was detected, in B ***p<0.001. ROI-image size 500x500 pixels taken with 10x objective. [file 13287_2024_3672_MOESM5_ESM.tif]

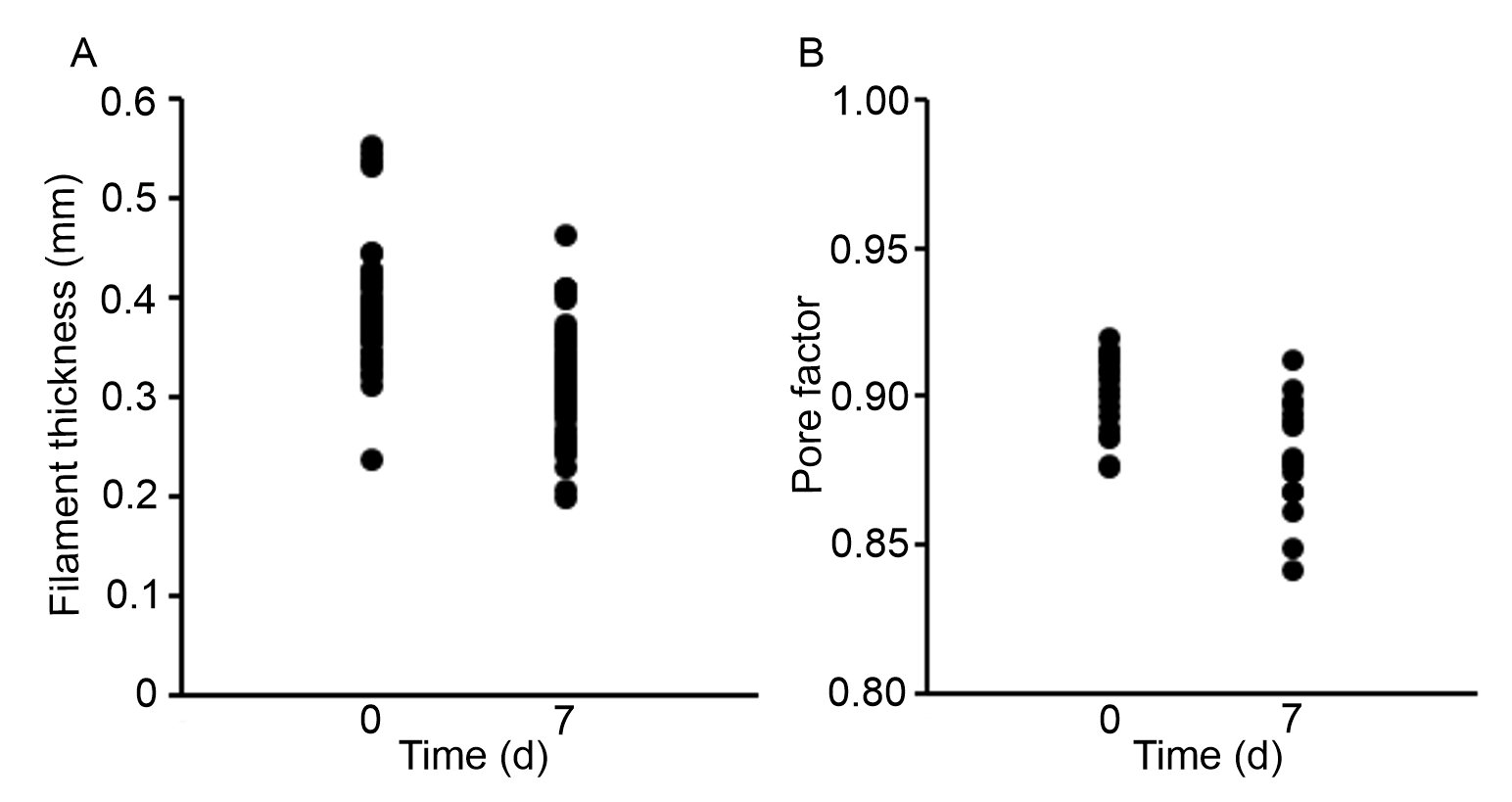

Supplement: Supplementary file 7 — Additional file 7 Fig. S10 Representative images of the printed structures for bioprinting hPSC-CEnCs. A For printing cells, line distance of 0.30 mm was used, resulting in filament fusion and uniform bioink layer in the first layer. B Bioprinted structure after 2nd printed layer imaged with a high-definition CCD-camera attached to the dispenser head mount immediately after printing. Scale bars 10 mm. C A representative two-layered bioprinted structure used for hPSC-CEnCs printing on a cell culture dish [file 13287_2024_3672_MOESM7_ESM.tif]

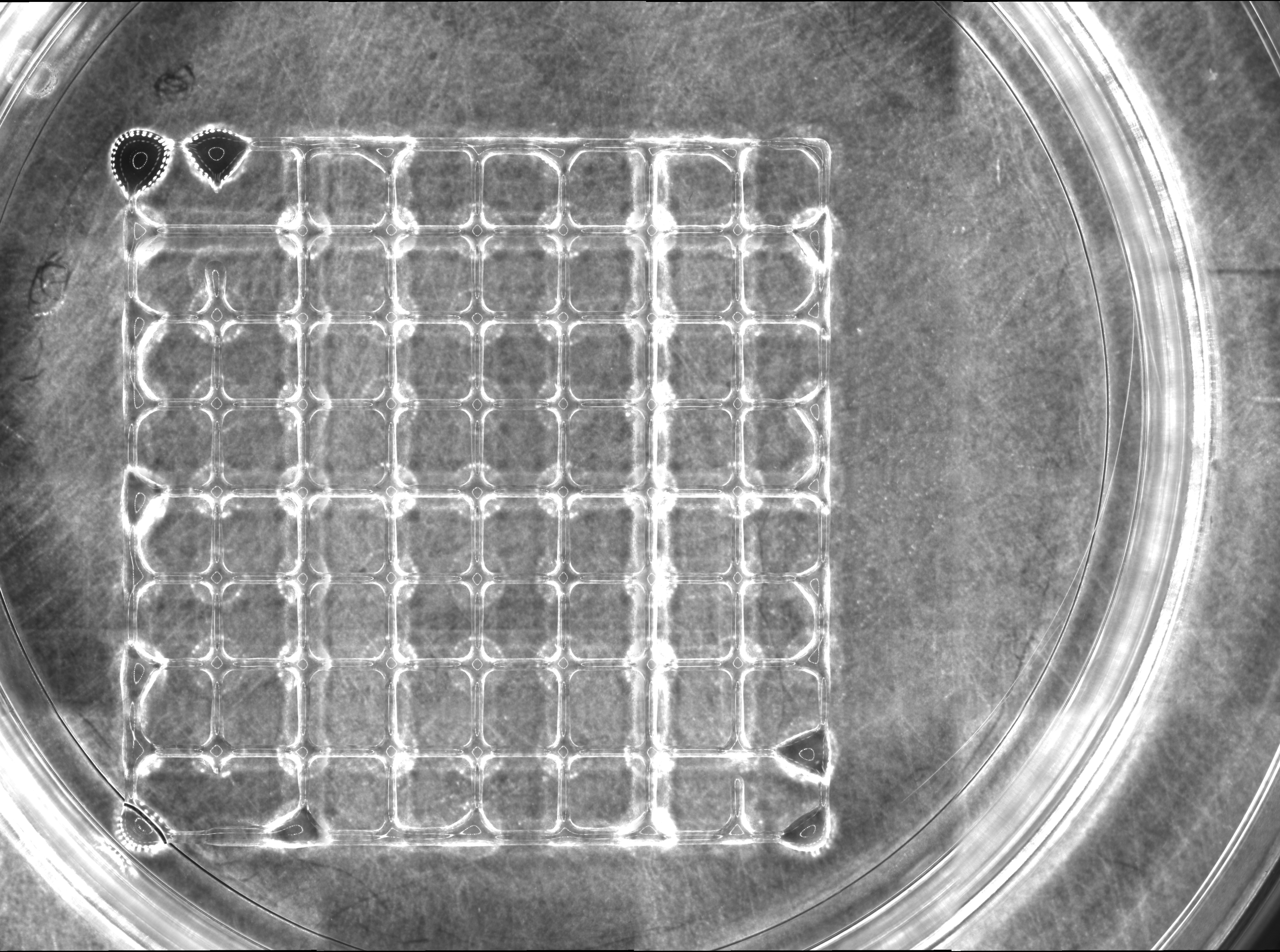

Supplement: Supplementary file 8 — Additional file 8 Fig. S11 Additional representative immunofluorescence stainings of bioprinted hPSC-CEnCs from day 1 (n=1) and day 7 (n=2) stained with ZO-1 (green), CD166 (red) and Hoechst (blue). Experiments conducted with the used hESC line. Scale bar 200 μm. [file 13287_2024_3672_MOESM8_ESM.tif]

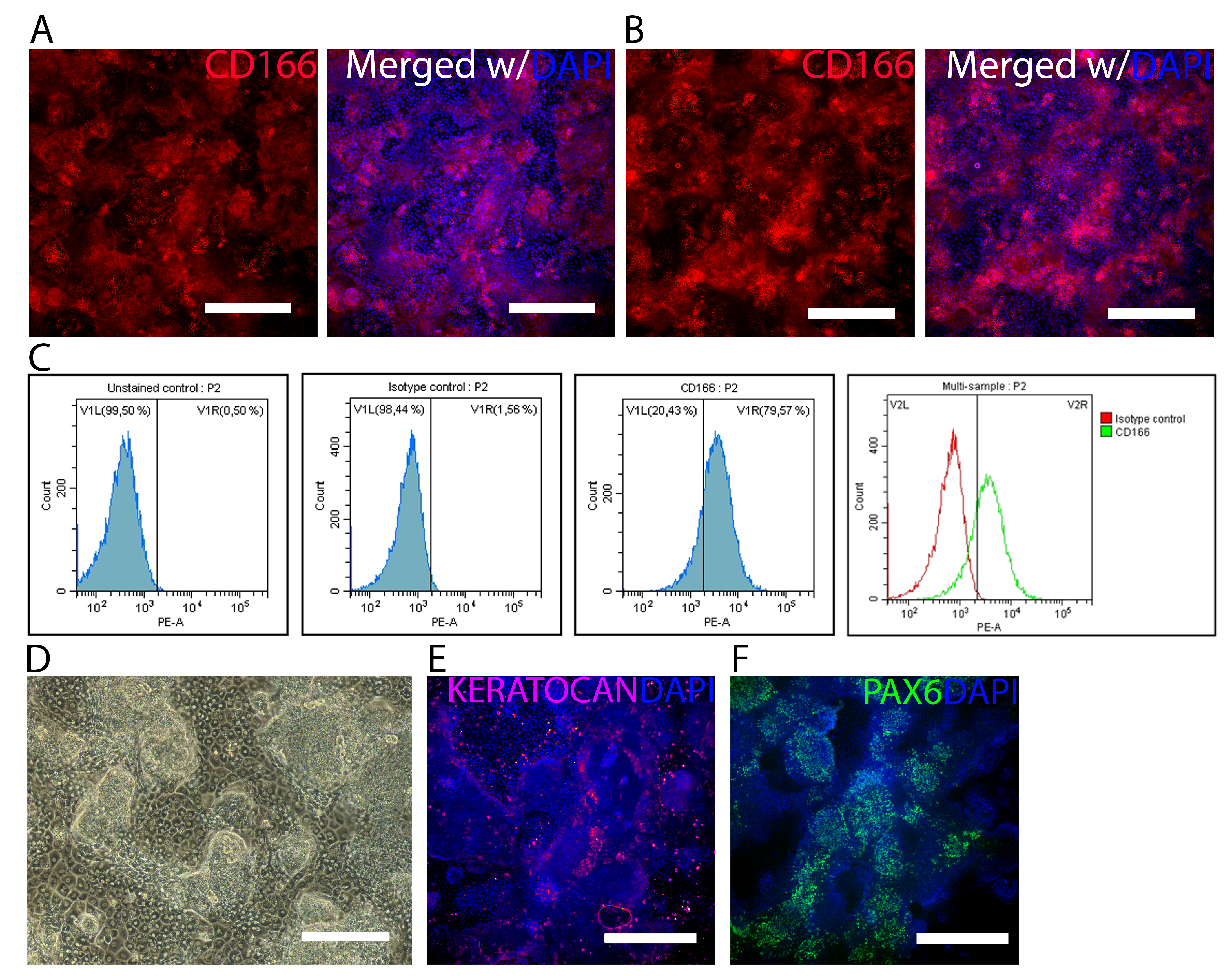

Supplement: Supplementary file 9 — Additional file 9 Fig. S12 Representative immunofluorescence images of bioprinted A and seeded B hPSC-CEnCs on fibrin membrane at day 7 (n=2). Experiments conducted with the used hESC line. Cell cultures are showing similar characteristics with ZO-1 and CD166 markers. C Immunofluorescence stainings of proliferation marker Ki67 shows growth of unwanted cells in the hPSC-CEnC culture in the bioprinted 7 days after bioprinting (n=1) and D also in the seeded hPSC-CEnC control on fibrin membrane (n=1). In A-B) ZO-1 (green), CD166 (red) and Hoechst (blue). Scale bars 100 μm, magnified image 50μm. In C–D Ki67 (green) Hoechst (blue). Scale bars 400 μm. [file 13287_2024_3672_MOESM9_ESM.tif]

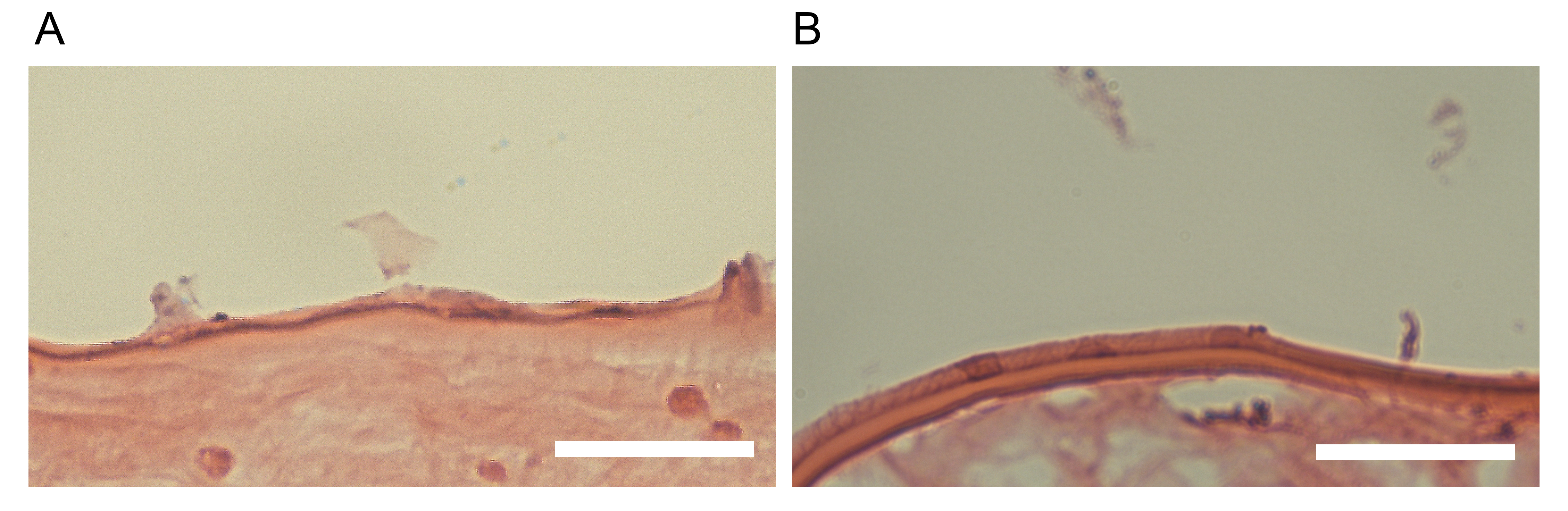

Supplement: Supplementary file 11 — Additional file 11 Fig. S2 A representative original image used for the printability and shape fidelity analysis of the bioink. [file 13287_2024_3672_MOESM11_ESM.tif]
